# TLN1 interacts with NGFR and suppresses the development of castration-resistant prostate cancer by upregulating NGFR

**DOI:** 10.3389/fimmu.2026.1802129

**Published:** 2026-04-23

**Authors:** Sixin Li, Anjie Chen, Chen Guo, Chenwei Gu, Zhonghao Tang, Si Shen, Dongjie Yang, Yuanyuan Mi

**Affiliations:** 1 Department of Urology, Affiliated Hospital of Jiangnan University, Wuxi, Jiangsu, China; 2 Wuxi School of Medicine, Jiangnan University, Wuxi, Jiangsu, China; 3 Department of Pathology, Affiliated Hospital of Jiangnan University, Wuxi, Jiangsu, China

**Keywords:** castration-resistant prostate cancer, NGFR, prostate cancer, therapeutic target, TLN1

## Abstract

**Background:**

Prostate cancer (PCa) is a common malignant tumor in males, and castration-resistant prostate cancer (CRPC) represents an advanced stage with limited treatment options and poor prognosis. Talin-1 (TLN1) is a cytoskeletal protein implicated in tumor progression, but its specific role and mechanism in CRPC remain unclear.

**Methods:**

Mass spectrometry (MS) was used to analyze serum peptides from patients with hormone-sensitive prostate cancer (HSPC) and CRPC. TLN1 expression was further validated in clinical prostate tissue samples (59 PCa, 17 benign prostatic hyperplasia) via immunohistochemistry, qPCR, and Western blot. Functional assays (CCK-8, colony formation, wound healing, Transwell) and a nude mouse xenograft model were employed to assess the effects of TLN1 knockdown on CRPC cell lines (DU145, PC3). Transcriptome sequencing, molecular docking, and co-immunoprecipitation (Co-IP) were conducted to explore downstream mechanisms and interactions. Western blot analysis was applied to examine the impact of TLN1 knockdown on apoptosis and the PI3K-AKT, MAPK, and NF-κB signaling pathways in CRPC cell lines. Rescue experiments were performed by knocking down both TLN1 and nerve growth factor receptor (NGFR).

**Results:**

TLN1 expression was significantly upregulated in CRPC patient serum and PCa tissues. Knockdown of TLN1 inhibited proliferation, migration, invasion, and epithelial-mesenchymal transition (EMT), promoted apoptosis in CRPC cells, and suppressed tumor growth *in vivo*. Transcriptome analysis identified NGFR as significantly upregulated upon TLN1 knockdown. TLN1 knockdown can influence the malignant progression of CRPC through the MAPK and PI3K-AKT signaling pathways. Molecular docking and Co-IP confirmed a direct interaction between TLN1 and NGFR. Knockdown of NGFR reversed the tumor-suppressive effects induced by TLN1 silencing.

**Conclusions:**

TLN1 inhibits the progression of CRPC by interacting with and regulating the tumor suppressor NGFR. The TLN1/NGFR axis represents a novel potential therapeutic target for CRPC.

## Introduction

Prostate cancer (PCa) is among the most prevalent malignant neoplasms affecting the male population, with recent years witnessing a marked escalation in both its incidence and associated mortality rates (1). Early diagnosis and therapeutic interventions, such as prostate-specific antigen screening and androgen deprivation therapy (ADT), have proven to be highly efficacious. In scenarios where surgical or radiotherapeutic options are not viable, ADT is employed as the principal therapeutic strategy. ADT functions by diminishing androgen levels, primarily testosterone, thereby inhibiting the proliferation of PCa cells. Given that androgens are pivotal in promoting the growth of these cells, their reduction can decelerate or impede disease progression. Nonetheless, PCa cells frequently develop resistance to ADT over time (2, 3). The majority of patients undergoing ADT eventually progress to castration-resistant prostate cancer (CRPC) within a period of 2–3 years (4, 5). The development of CRPC is attributed to the capacity of certain PCa cells to sustain proliferation even in a low-androgen milieu. The persistent progression of PCa can be attributed to various mechanisms, including gene mutations that induce structural alterations and hyperactivation of the androgen receptor, overexpression of the androgen receptor, and the activation of alternative signaling pathways (5–7). As PCa advances to CRPC and loses its androgen dependence, ADT frequently becomes ineffective. CRPC is typically indicative of an advanced disease stage, characterized by a poor patient prognosis and consistently low five-year survival rates (8, 9). A study found that prior to the initiation of ADT, there exists a subset of PCa cells with intrinsic castration-resistant characteristics in untreated PCa. These pre-existing castration-resistant cells can rapidly expand during ADT and drive the development of CRPC (10). The study further indicates that CRPC-like cells in primary PCa are fully developed castration-resistant cells. Immunohistochemistry staining results show that such cells are highly enriched in locally recurrent CRPC and metastatic castration-resistant prostate cancer (mCRPC). This suggests that CRPC-like cells already present in early-stage PCa may be selected and expanded as the dominant clone under androgen-deprived conditions, ultimately leading to disease progression. The majority of patients with metastatic PCa inevitably progress to CRPC within 14 to 30 months of initiating ADT, with median survival generally not surpassing two years (11). Currently, the management of CRPC poses a significant clinical challenge.

Talin-1 (TLN1), encoded by the *TLN1* gene, is a cytoskeletal protein that plays a critical role in focal adhesion (12–14) and integrin activation (15, 16) in normal cells, indicating its potential involvement in tumor migration, invasion, and metastasis (17). Elevated levels of TLN1 have been demonstrated to enhance the invasiveness and migratory capabilities of several malignancies, including glioblastoma, PCa, and nasopharyngeal carcinoma. However, an inverse role has been observed in hepatocellular carcinoma (18–21). Reduced expression of TLN1 has been associated with increased invasion and metastasis in two cancer types and is linked to liver metastasis, TNM stage, and lymph node metastasis in colorectal cancer (22, 23). TLN1 has been recognized as a prognostic biomarker in nasopharyngeal carcinoma, oral squamous cell carcinoma, triple-negative breast cancer, colorectal cancer, myeloid leukemia, and ovarian serous carcinoma, and is correlated with poor prognosis in oral squamous cell carcinoma, triple-negative breast cancer, and ovarian serous carcinoma (18, 24–28). Furthermore, Jin et al. (19) have elucidated that cdk5-mediated phosphorylation of TLN1 facilitates bone metastasis in PCa through the activation of β1 integrin. Xu et al. (29) demonstrated that increased expression of TLN1 is correlated with malignancy and lymph node metastasis in PCa. Furthermore, elevated TLN1 levels, when considered alongside a Gleason score exceeding 7, enhance the prediction of lymph node metastasis in patients with PCa. Although it is clear that TLN1 is a critical factor in PCa, research focusing on TLN1, especially in the context of CRPC, remains sparse.

Prior to initiating this project, we performed mass spectrometry (MS) analysis to compare the peptide expression profiles in serum samples from patients with hormone-sensitive prostate cancer (HSPC) and those with CRPC. The results revealed that TLN1 was upregulated in the serum of CRPC patients. Further examination of clinical tissue samples confirmed that TLN1 expression is elevated in PCa tissues. Based on these findings, we selected the hormone-insensitive PCa cell lines DU145 and PC3 for subsequent phenotypic experiments.

**Figure 1:**
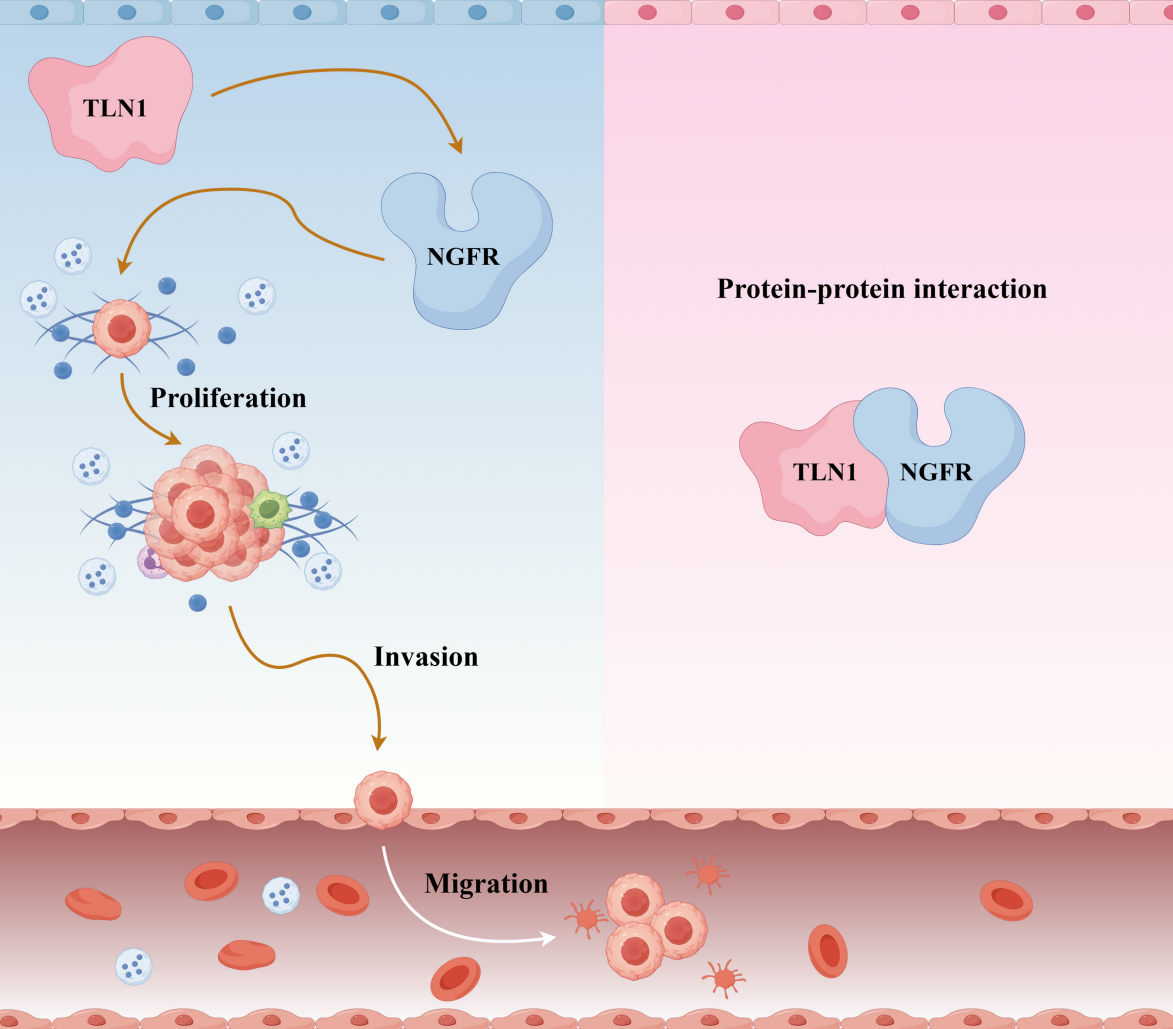
Mechanism diagram of TLN1 influencing the progression of CRPC. TLN1 interacts with NGFR and suppresses the development of CRPC by regulating NGFR.

The experimental results demonstrated that TLN1 can interact with the nerve growth factor receptor (NGFR). In addition, knockdown of TLN1 suppressed the proliferation, invasion, and migration of CRPC cells by upregulating NGFR expression (**Figure 1**).

## Materials and methods

### Cell culture

PC3 and DU145 human PCa cell lines and a human prostatic cell line (RWPE-1) were obtained from the Chinese Academy of Sciences Cell Bank. PC3 cells were grown in RPMI-1640, and DU145 and RWPE-1 in high-glucose DMEM, both with 10% FBS, 100 µg/mL streptomycin, and 100 U/mL penicillin. Cultures were maintained at 37°C with 5% CO2.

### Human tissues

Paraffin-embedded tissue blocks were procured from the Department of Pathology at the Affiliated Hospital of Jiangnan University between January 2020 and December 2022. These samples comprised specimens from 59 patients diagnosed with PCa and 17 patients with benign prostatic hyperplasia (BPH). Importantly, none of the patients had received ADT or radiotherapy prior to surgical intervention. The collection and use of clinical samples were sanctioned by the Research Ethics Committee of the Affiliated Hospital of Jiangnan University (Approval No. LS202128). We affirm that all procedures adhered strictly to the guidelines and regulations established by the Research Ethics Committee of the Affiliated Hospital of Jiangnan University.

### Quantitative real-time PCR

Total RNA was isolated utilizing the FastPure Cell/Tissue Total RNA Isolation Kit V2 (Vazyme, China). Subsequently, the RNA was reverse transcribed into complementary DNA (cDNA) using the HiScript III RT SuperMix for qPCR (+gDNA wiper) (Vazyme, China), following the manufacturer’s protocol. Primer sequences for the TLN1, NGFR and GAPDH genes are detailed in **Supplementary Table 1**. The SYBR Green Real-Time Quantitative PCR Detection Kit (Vazyme, China) was employed for PCR amplification on an ABI 7500 PCR system (Applied Biosystems, USA). The qPCR protocol initiated at 95°C for 30 seconds, followed by 40 cycles of 95°C for 10 seconds and 60°C for 30 seconds. All qPCR experiments used GAPDH as the housekeeping gene. Normalization of qPCR data is commonly performed using the ΔΔCt method. This approach ensures comparability through dual calibration: first, the Ct value of the target gene is subtracted by the Ct value of a reference gene (GAPDH) to obtain ΔCt, correcting for variations in sample loading and RNA quality. Next, the ΔCt of each sample is subtracted by the average ΔCt of the control group to yield ΔΔCt. Finally, the fold change in expression of the target gene relative to the control is calculated using the formula 2^(-ΔΔCt). All experiments were conducted in triplicate.

### Immunohistochemistry

Tissue samples were procured from the Affiliated Hospital of Jiangnan University. The tissue sections underwent fixation in 10% formalin, were subsequently embedded in paraffin, and sectioned into 4µm slices. Following deparaffinization and rehydration, antigen retrieval was conducted in citrate buffer (pH 6.0) utilizing microwave heating. The sections were then blocked with 5% bovine serum albumin (BSA) and incubated overnight at 4°C with a TLN1 polyclonal rabbit antibody at a 1:500 dilution (catalog number 14168-1-AP, Proteintech, Chicago, USA). After washing with phosphate-buffered saline (PBS), a horseradish peroxidase-conjugated (HRP) goat anti-rabbit secondary antibody at a 1:2000 dilution (catalog number ab205718, Abcam, Cambridge, England) was applied at room temperature for 2 hours. Counterstaining was performed using DAB chromogen and hematoxylin. Microscopic imaging was conducted at magnifications of 100X and 200X using a standard microscope. Quantitative analysis of the data was carried out using ImageJ software.

### Cell transfection

Plasmid transfection TLN1 and NGFR small interference RNA designed by (https://www.genechem.com.cn/proinfo/20.html), using the scramble sequence of nonsense as a negative control (NC) group. The efficiency of infection was measured using qPCR. Green fluorescent protein fluorescence showed that the transfection efficiency was more than 80%. Here are some details about all sequences (**Supplementary Table 2**).

### Cell-counting kit 8 assay

Cell viability was assessed using the CCK-8 assay kit (Beyotime Institute of Biotechnology). Cells (8,000 cells/mL) were plated at 100 μL per well in 96-well plates. At intervals of 0, 1, 2, 3, and 4 days post-seeding, 10 µL of CCK-8 solution was added to each well. Following an incubation period of three hours, absorbance was measured at 450 nm using a microplate reader. All experimental procedures were conducted in triplicate to ensure reproducibility.

### Colony formation assay

Following the specified transfection procedure, the PCa cells were plated in six-well culture dishes, with each experimental condition being replicated in three wells. Cells were seeded in 6-well plates at a density of 1,000 cells per well in 2 ml of complete medium. The cells were maintained under observation for a duration of 14 days, during which the culture medium was replenished every three days. Subsequently, the cell colonies were fixed using paraformaldehyde (1 mL per well; sourced from Shanghai Pharmaceutical Group, Shanghai, China) for a period ranging from 30 to 60 minutes. This was followed by staining with 5% crystal violet for 20 minutes. Quantitative data analysis was conducted utilizing ImageJ software.

### Tumor formation assay in nude-mouse models

A cohort of five 4-week-old male BALB/c nude mice, procured from Beijing HFK Bioscience Co. Ltd., was randomly allocated into two experimental groups. DU145 cells, transfected with either shCtrl or shTLN1-1, were subcutaneously injected into each mouse at a concentration of 2×10^7 cells per mouse. DU145 cells (2×10^7) were resuspended in 50 µL of PBS and mixed with an equal volume of Matrigel (50 µL). The resulting cell suspension (100 µL total volume, at a concentration of 2×10^8 cells/mL) was subcutaneously injected into each mouse. Tumor progression was assessed every five days by measuring and calculating tumor volume with a vernier caliper. Tumor length (L, the longest diameter) and width (W, the longest diameter perpendicular to L) were measured. Tumor volume was calculated using the formula V = 0.5×L×W×W. Before the 28th day, the mice were anesthetized using isoflurane and subsequently euthanized via cervical dislocation. Tumors were excised for mass determination. The experimental protocol (JN. No20240830b0240315[402]) received approval from the Ethics Committee of Jiangnan University, and the animal research conducted in this study adheres to the ARRIVE guidelines.

### Transwell assay

To assess cell migration, 5×10^4 cells were seeded into the upper compartment of Transwell chambers (8pm pore size, Corning) containing 200 µL of serum-free DMEM medium. The lower compartment was supplemented with 750 µL of DMEM containing 20% fetal bovine serum (FBS). After a 24-hour incubation period, cells that had migrated to the lower chamber were fixed with 4% paraformaldehyde and subsequently stained with 5% crystal violet. The stained cells were then observed under a light microscope at 200X magnification.

### Wound healing assay

The knockdown and control group cells were cultured in six-well plates with media supplemented with 10% fetal bovine serum, with each group comprising three replicate wells. Upon reaching a cell confluence of 80-90%, a scratch was created at the left end of the center of each well using a scratch tester. The cells were subsequently washed three times with PBS. Images were captured at 0 hours and 24 hours post-scratch, and the extent of cell migration was quantified using ImageJ software.

### Western blot

Total protein extraction was performed using RIPA buffer (Beyotime). The extracted proteins were subsequently resolved on 8% SDS-PAGE gels and transferred onto PVDF membranes. Following a blocking step, the membranes were incubated overnight at 4°C with primary antibodies targeting TLN1 (dilution 1:20,000, 14168-1-AP, Proteintech, Chicago, USA), NGFR (dilution 1:10,000, BM4278, BOSTER, Wuhan, China), E-cadherin (dilution 1:5,000, 20874-1-AP, Proteintech, Chicago, USA), N-cadherin (dilution 1:6,000, 22018-1-AP, Proteintech, Chicago, USA), Caspase3 (dilution 1:1000, GB155600, Servicebio, Wuhan, China), Cleaved-Caspase-3 (dilution 1:1000, GB115733, Servicebio, Wuhan, China), Bcl2 (dilution 1:1000, GB154380, Servicebio, Wuhan, China), Bax (dilution 1:1000, GB15690, Servicebio, Wuhan, China), PI3K (dilution 1:2000, GB115763, Servicebio, Wuhan, China), p-PI3K (dilution 1:2000, GB150210, Servicebio, Wuhan, China), AKT (dilution 1:1000, GB15689, Servicebio, Wuhan, China), p-AKT (dilution 1:1000, GB150002, Servicebio, Wuhan, China), ERK (dilution 1:1000, GB15560, Servicebio, Wuhan, China), p-ERK (dilution 1:1000, GB120024, Servicebio, Wuhan, China), JNK (dilution 1:1000, GB154321, Servicebio, Wuhan, China), p-JNK (dilution 1:1000, GB12018, Servicebio, Wuhan, China), p38 (dilution 1:1000, GB154685, Servicebio, Wuhan, China), p-p38 (dilution 1:1000, GB153380, Servicebio, Wuhan, China), p65 (dilution 1:1000, GB15997, Servicebio, Wuhan, China), p-p65 (dilution 1:1000, GB153882, Servicebio, Wuhan, China), β-actin (dilution 1:3000, GB15003, Servicebio, Wuhan, China), and GAPDH (dilution 1:7,000, 10494-1-AP, Proteintech, Chicago, USA). This was followed by a 1-hour incubation at room temperature with a goat anti-rabbit secondary antibody conjugated to HRP. Enhanced chemiluminescence (ECL) was employed to visualize all blots. In Western blotting experiments for tumor expression validation, the grayscale value of the target protein band in each sample was normalized to that of its corresponding internal reference protein to compare the expression levels of the target gene across tumor samples.

### Transcriptome analysis

Initially, DU145 cells were transfected with plasmids extracted using the Trizol reagent (Invitrogen). An RNA library was subsequently constructed, and preliminary quantification was conducted using the Qubit 2.0 Fluorometer. Illumina sequencing was then carried out, producing 150bp paired-end reads. The image data from the sequencing fragments, obtained via high-throughput sequencing, were converted into sequence data through CASAVA base recognition, utilizing clean data. The reference genome and gene model annotation files were acquired from the genome database and aligned with the sequence data. Novel gene prediction was performed using StringTie (version 1.3.3b) as described by Mihaela Pertea et al. (2015), and read counts mapped to each gene were calculated using featureCounts (version 1.5.0-p3). The differential expression analysis between the two comparative groups, each comprising two biological replicates, was conducted using the DESeq2 software (version 1.16.1). Subsequently, the Gene Ontology (GO) enrichment analysis of the differentially expressed genes was performed utilizing the clusterProfiler software (version 3.4.4), which accounted for gene length bias. Protein-protein interaction (PPI) analysis of the differentially expressed genes was carried out based on the Search Tool for the Retrieval of Interacting Genes/Proteins (STRING) database, encompassing known and predicted interactions. Alternative splicing events were analyzed using the rMATS software (version 3.2.5), while the mutation sites within the sample data were examined using the GATK software (version 3.7). Variant site annotation was conducted with the SnpEff software (version 4.3q). Additionally, the Weighted Correlation Network Analysis (WGCNA) was employed to elucidate gene association patterns across different samples.

### Molecular docking

The proteins used for docking were TLN1 (UniProt ID: Q9Y490) and NGFR (UniProt ID: P08138), with structural models retrieved from the AlphaFold Protein Structure Database (https://alphafold.ebi.ac.uk/). Protein-protein molecular docking was performed using the HDOCK server (http://hdock.phys.hust.edu.cn/). The MM/GBSA module of the HawkDOCK server (http://cadd.zju.edu.cn/hawkdock/) was employed to calculate the binding free energy and rank residue-level contributions. The docking score served as the primary criterion for evaluating results, with the top-ranked model among ten output conformations selected for further analysis. Finally, we used PyMOL 2.4 to visualize high-affinity binding sites between the proteins, providing a clear representation of their interaction.

### Co-immunoprecipitation assay

The experiment was conducted utilizing DU145 and PC3 cells. A suitable TLN1 primary antibody was introduced to the cell lysate to facilitate its binding to Protein A Sepharose. Based on the concentration of expressed proteins and cell lysate, 1-5 µg of antibody and 10 µL of Protein A (at a 50% suspension) were combined to achieve a total volume of 1mL, and the mixture was incubated at 4°C for 4 hours. The beads were collected by centrifugation at 10,000 rpm for 30 seconds and subsequently washed three times with 1X HNTG buffer. The precipitate was eluted using 30 µL of 1X Laemmli loading buffer. The interaction between TLN1 and NGFR was analyzed through sodium dodecyl sulfate-polyacrylamide gel electrophoresis (SDS-PAGE) and western blotting, following a heating step at 100°C for 10 minutes.

### Polypeptide extraction and mass spectrometry

For the extraction of serum peptides, samples were initially placed in a well plate. To each sample, 10 µL of magnetic bead binding buffer was added, followed by the addition of 10 µL of plasma. The resulting mixture was incubated at room temperature. Subsequently, the plate was positioned on a magnetic separator for one minute to facilitate the enrichment of beads at the bottom and sides of the wells, thereby separating them from the supernatant. After the supernatant was removed, the samples were returned to the plate. Next, 100 µL of magnetic bead wash buffer was added to each sample, and the separation step was repeated, followed by the removal of the supernatant. Finally, 10µL of magnetic bead elution buffer was added to each sample in the plate. After incubation, the supernatant was removed, and the eluate was collected for measurement. MS analysis was conducted in accordance with the protocol outlined by Mun et al. (30). Through comparison with a protein-coding database, 1,084 peptides exceeding 10 kDa in size were identified. Based on the “quality” and “significance” criteria suggested by the MS sequencing provider, genes exhibiting over a 2-fold variation in expression and potentially implicated in tumor regulation were selected for subsequent analysis.

### Statistical analysis

Prior to conducting statistical analyses, the data distribution was normalized. Group comparisons were performed using the t-test. Statistical analyses were executed using SPSS version 17.0, with P-values less than 0.05 deemed statistically significant. Data are presented as the mean ± standard deviation. Visual representations were generated using GraphPad Prism 10 and Adobe Illustrator CC 2018.

## Results

### TLN1 was highly expressed in PCa tissue compared to normal prostate tissue

**Figure 2:**
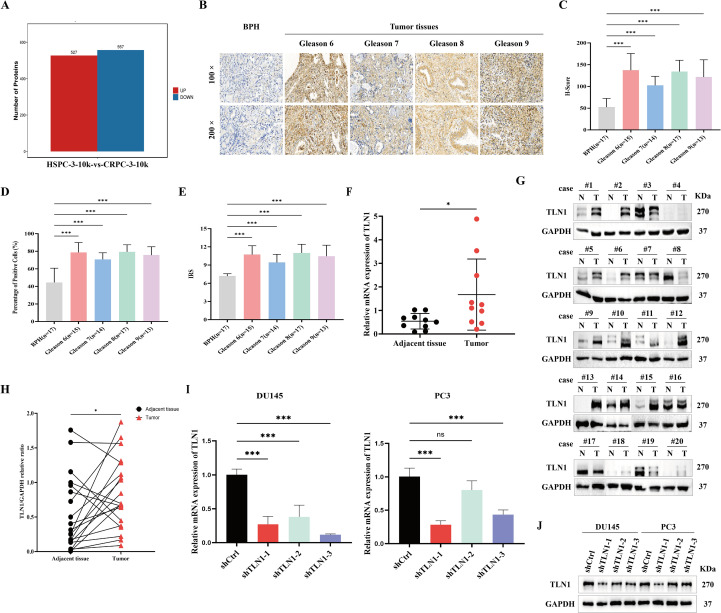
TLN1 was highly expressed in PCa compared to normal prostate tissue. **(A)** MS analysis revealed differential peptide expression in the serum of patients with CRPC and HSPC, identifying 527 peptides with increased expression and 557 peptides with decreased expression. **(B)** Expression of TLN1 in BPH and PCa tissues with different Gleason scores was detected by immunohistochemistry. **(C–E)** Quantitative analysis of immunohistochemical results using H-score, positive cell percentage, and immunoreactivity score showed that TLN1 expression was significantly higher in PCa tissues than in BPH tissues. **(F)** TLN1 mRNA expression levels in 10 pairs of PCa and adjacent normal tissues were detected by qPCR. **(G, H)** TLN1 protein expression levels in 20 pairs of PCa and adjacent normal tissues were detected by Western blotting. **(I, J)** Knockdown efficiency of three TLN1-targeting plasmids was validated by qPCR and Western blotting, respectively; shCtrl served as the negative control, while shTLN1-1, shTLN1-2, and shTLN1–3 represent the TLN1 knockdown plasmids. Data were presented as mean with standard deviation. "ns" indicates a P-value > 0.05. *P < 0.05 and ***P < 0.001.

Among the 1,084 peptide segments identified by MS in serum samples from patients with HSPC and CRPC (**Figure 2A**), we selected 23 candidate genes, including TLN1, based on screening criteria of more than a 2-fold difference in expression and potential involvement in tumor regulation. First, we examined the expression of TLN1 in PCa tissues. Immunohistochemical analysis was performed on 17 BPH samples and 59 PCa samples to assess TLN1 expression intensity. The PCa tissues were categorized into four groups according to the Gleason score: Gleason 6 (n=15), Gleason 7 (n=14), Gleason 8 (n=17), and Gleason 9 (n=13). Although no significant correlation was observed between TLN1 expression and the Gleason score, the results indicated that TLN1 expression was significantly higher in PCa tissues compared to BPH tissues (**Figures 2B–E**). Furthermore, we analyzed TLN1 expression in 10 paired and 20 paired clinical samples of PCa and adjacent normal tissues using qPCR and Western blot, respectively. These experiments confirmed that TLN1 is indeed highly expressed in PCa (**Figures 2F–H**).

Prior to investigating the functional role of TLN1 in PCa, we constructed three TLN1 knockdown plasmids. The knockdown efficiency was evaluated in the PCa cell lines DU145 and PC3 via qPCR and Western blot. The results demonstrated that shTLN1–1 exhibited the most effective knockdown (**Figures 2I, J**).

### The downregulation of TLN1 inhibits the development of CRPC in both *in vivo* and *in vitro*

**Figure 3:**
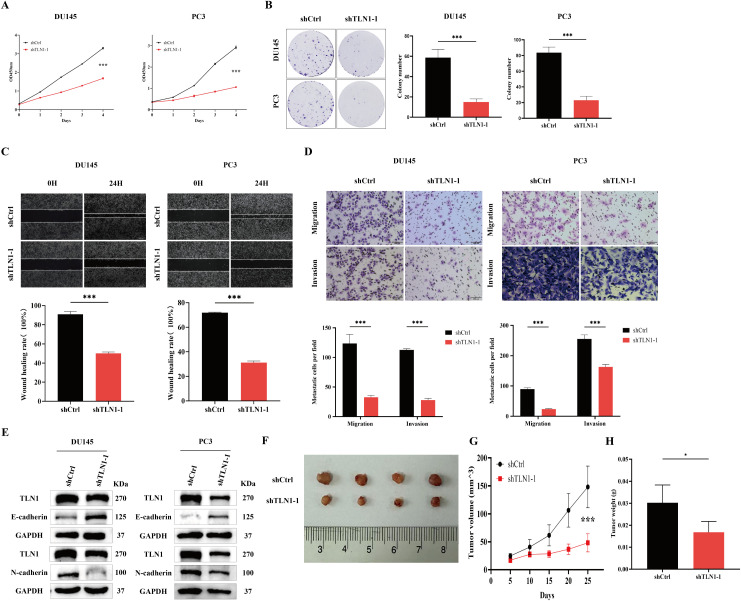
The downregulation of TLN1 inhibits the development of CRPC in both *in vivo* and *in vitro*. **(A)** CCK-8 assay results demonstrate that knockdown of TLN1 significantly inhibits the proliferation of CRPC cell lines DU145 and PC3. The graph shows changes in absorbance at 450 nm measured from day 1 to day 4. **(B)** Colony formation assay indicates that knockdown of TLN1 markedly suppresses the colony-forming ability of DU145 and PC3 cells. **(C)** Wound healing assay reveals that knockdown of TLN1 significantly impairs the migration and wound closure capacity of DU145 and PC3 cells. **(D)** Transwell assay shows that knockdown of TLN1 notably reduces the migration and invasion abilities of DU145 and PC3 cells. **(E)** Western blot analysis indicates that knockdown of TLN1 upregulates the epithelial marker E-cadherin and downregulates the mesenchymal marker N-cadherin. **(F)** Results of the tumor xenograft experiment in nude mice. **(G)** Showed xenograft tumor growth curve and **(H)** illustrated tumor weight measurements. Data were presented as mean with standard deviation. *P < 0.05 and ***P < 0.001.

Before proceeding to *in vivo* experiments, we first conducted a series of *in vitro* assays to investigate the functional impact of TLN1 knockdown on PCa cell lines. CCK-8 assay results showed that TLN1 knockdown significantly inhibited the proliferation of both DU145 and PC3 cells compared to the shCtrl group (**Figure 3A**). Colony formation assays further demonstrated that downregulation of TLN1 markedly reduced the number of colonies formed by both cell lines (**Figure 3B**). Additionally, wound healing and Transwell assays revealed that TLN1 knockdown suppressed both migratory and invasive capabilities of the cells (**Figures 3C, D**). In the assessment of epithelial-mesenchymal transition (EMT) markers, the shTLN1–1 group exhibited upregulation of the epithelial marker E-cadherin and downregulation of the mesenchymal marker N-cadherin, indicating that TLN1 downregulation inhibits the EMT process in DU145 and PC3 cells (**Figure 3E**).

Subsequently, we established a mouse xenograft model to validate the role of TLN1 *in vivo*. DU145 cells treated with shTLN1–1 or shCtrl were subcutaneously inoculated into the flanks of mice. On day 25, tumors derived from shTLN1–1 cells showed significantly reduced weight and volume compared to those from the shCtrl group (**Figures 3F–H**). These results demonstrate that TLN1 downregulation suppresses malignant progression of CRPC both *in vitro* and *in vivo*.

### The exploration of the potential regulatory mechanisms of TLN1 in CRPC

**Figure 4:**
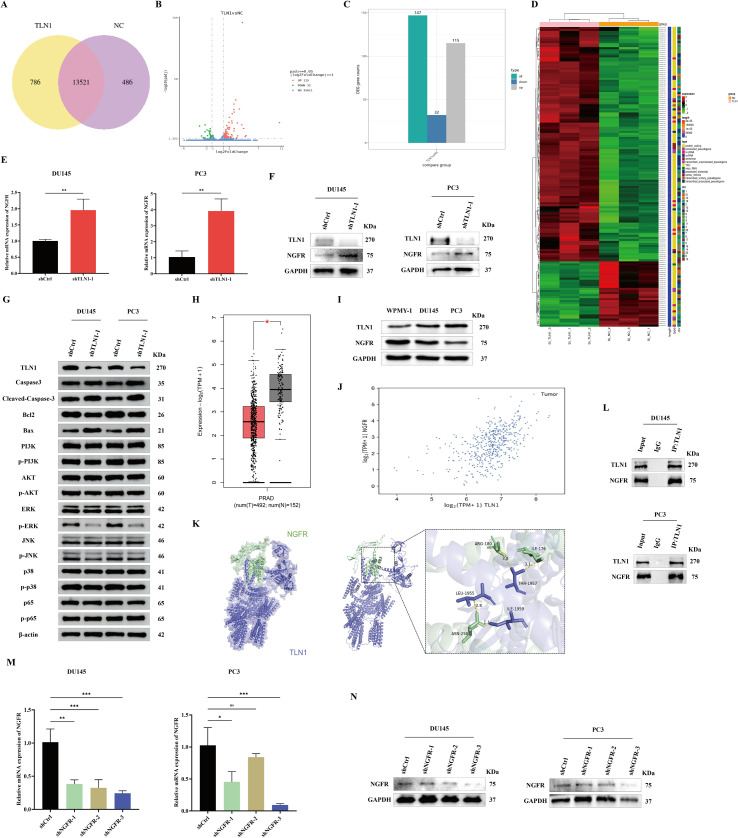
The exploration of the potential regulatory mechanisms of TLN1 in CRPC. **(A)** RNA sequencing was performed in DU145 cells, comparing the number of protein-coding genes detected between the TLN1 knockdown group and the control group. **(B)** Volcano plot of differentially expressed genes, illustrating the changes in gene expression between TLN1 knockdown and control groups in DU145 cells. **(C)** Statistics of differentially expressed genes under TLN1 knockdown versus control conditions in DU145 cells. **(D)** Heatmap of differentially expressed genes, with color gradient representing gene expression levels—red indicates high expression and green indicates low expression. **(E, F)** NGFR upregulation at the mRNA and protein levels after TLN1 knockdown was verified by qPCR and Western blot, respectively. **(G)** Western blot analysis was employed to assess the effects of TLN1 knockdown on apoptosis and the PI3K-AKT, MAPK, and NF-κB signaling pathways in DU145 and PC3 cell lines. **(H)** Expression profile of NGFR in PCa tissues based on analysis from the GEPIA2 database. **(I)** Western blot analysis was conducted to assess the expression of TLN1 and NGFR in the normal prostate cell line WPMY-1 and the CRPC cell lines DU145 and PC3. **(J)** Correlation analysis of TLN1 and NGFR expression in PCa was performed using the GEPIA3 database. **(K)** Molecular docking simulation results of TLN1 with NGFR. **(L)** Co-IP assay confirming the protein interaction between TLN1 and NGFR. **(M, N)** Knockdown efficiency of three NGFR-targeting plasmids was evaluated by qPCR and Western blot, respectively; shCtrl served as the negative control, while shNGFR-1, shNGFR-2, and shNGFR-3 represent NGFR-specific knockdown plasmids. Data were presented as mean with standard deviation. *P < 0.05, **P < 0.01 and ***P < 0.001.

To further investigate the regulatory mechanism of TLN1 in PCa, we performed RNA sequencing on DU145 cells treated with shTLN1–1 and shCtrl. The results showed that 13,521 genes were commonly expressed between the two groups (**Figure 4A**), among which 147 genes exhibited significant differences: 115 genes were significantly upregulated and 32 genes were significantly downregulated (**Figures 4B, C**). From these, we selected NGFR, a significantly upregulated gene (padj < 0.05, log_2_FoldChange > 1.5), as a candidate downstream target for further study (**Figure 4D**). Validation at both the mRNA and protein levels confirmed that NGFR expression increased upon TLN1 knockdown, consistent with the sequencing results (**Figures 4E, F**). Western blot was also conducted to examine whether TLN1 influences the malignant progression of CRPC through apoptosis−related molecules (Caspase3, Cleaved−Caspase−3, Bax, Bcl2) and key signaling pathways including PI3K−AKT, MAPK, and NF−κB. The results showed that TLN1 knockdown up−regulated the pro−apoptotic proteins Bax and Cleaved−Caspase−3 and down−regulated the anti−apoptotic protein Bcl2, indicating that TLN1 knockdown promotes apoptosis in CRPC cell lines. Moreover, TLN1 knockdown reduced the phosphorylation of AKT, ERK, and JNK, suggesting that TLN1 suppresses the malignant progression of CRPC by inhibiting phosphorylation of AKT in the PI3K−AKT pathway and of ERK and JNK in the MAPK pathway (**Figure 4G**). Furthermore, analysis of the GEPIA2 database revealed that NGFR expression was significantly lower in PCa tissues compared to adjacent normal tissues (*P* < 0.05) (**Figure 4H**), and previous studies have suggested that NGFR may function as a tumor suppressor in PCa. The expression of TLN1 and NGFR was also examined in the normal human prostate cell line WPMY−1 and the CRPC cell lines DU145 and PC3. Consistent with previous findings, TLN1 was highly expressed in CRPC cell lines, while NGFR expression was lower compared to the normal prostate cell line WPMY−1 (**Figure 4I**). Subsequently, correlation analysis of TLN1 and NGFR expression in PCa was performed using the GEPIA3 database. The results revealed a positive correlation between TLN1 and NGFR expression in PCa (r=0.506, *p*-value=1.2e-33) (**Figure 4J**). To predict whether TLN1 interacts directly with NGFR, we performed molecular docking simulations (**Figure 4K**) and subsequently confirmed through Co-IP experiments that TLN1 does indeed interact with NGFR (**Figure 4L**). We then constructed three NGFR knockdown plasmids and, after verifying their knockdown efficiency, selected shNGFR-3 for subsequent phenotypic experiments in CRPC (**Figures 4M, N**).

### Downregulation of NGFR promotes CRPC development *in vitro*

**Figure 5:**
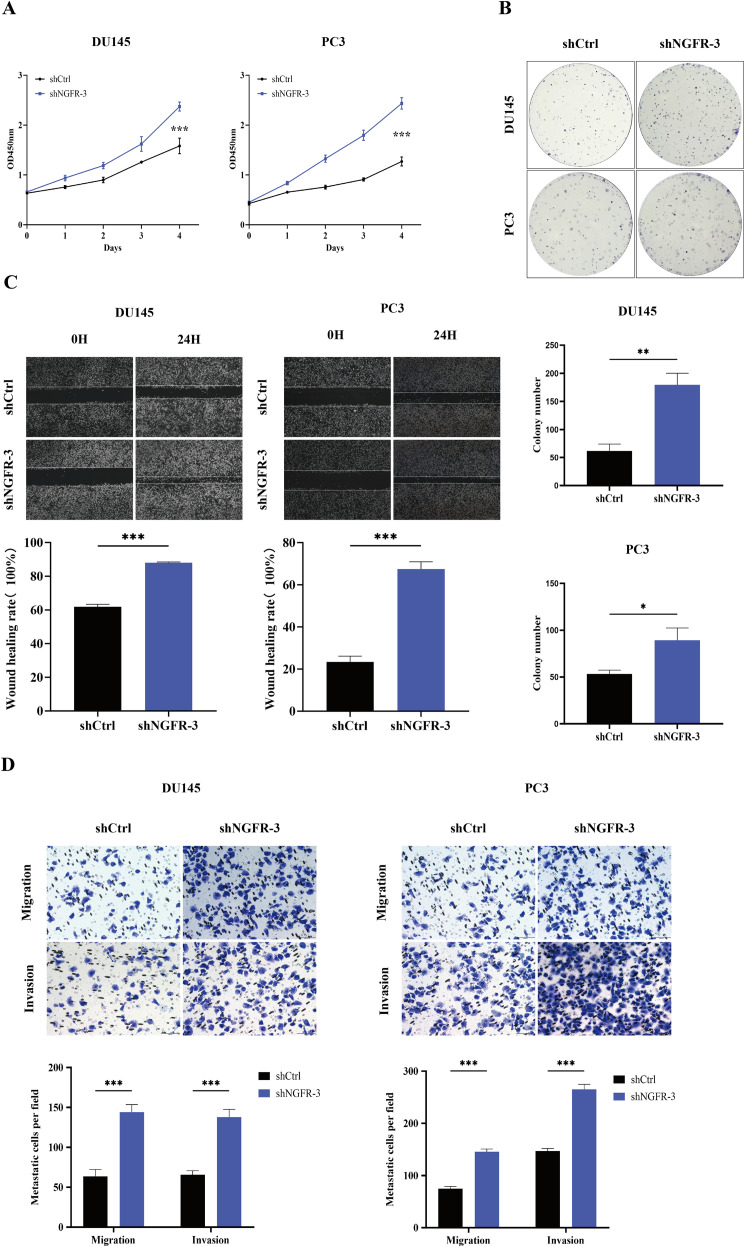
Downregulation of NGFR promotes CRPC development *in vitro*. **(A)** CCK-8 assay demonstrates that knockdown of NGFR significantly promotes the proliferation of CRPC cell lines DU145 and PC3. The graph shows the changes in absorbance at 450 nm measured from day 1 to day 4. **(B)** Colony formation assay indicates that knockdown of NGFR markedly enhances the colony-forming ability of DU145 and PC3 cells. **(C)** Wound healing assay reveals that knockdown of NGFR significantly promotes the migration and wound closure capacity of DU145 and PC3 cells. **(D)** Transwell assay shows that knockdown of NGFR notably increases the migration and invasion abilities of DU145 and PC3 cells. Data were presented as mean with standard deviation. *P < 0.05, **P < 0.01 and ***P < 0.001.

We systematically evaluated the effect of NGFR on the malignant phenotype of PCa cells using CCK-8, colony formation, wound healing, and Transwell assays. The CCK-8 assay showed that shNGFR-3 treatment significantly promoted the proliferation of both DU145 and PC3 cells compared to the shCtrl group (**Figure 5A**). Colony formation assays further demonstrated that NGFR knockdown markedly increased the number of colonies formed by both cell lines (**Figure 5B**). In addition, wound healing and Transwell assays revealed that NGFR knockdown significantly enhanced the migratory and invasive capabilities of DU145 and PC3 cells (**Figures 5C, D**). These consistent results indicate that NGFR downregulation promotes the proliferation, migration, and invasion of CRPC cells *in vitro*.

### TLN1 downregulation inhibits the occurrence and development of CRPC by upregulating NGFR

**Figure 6:**
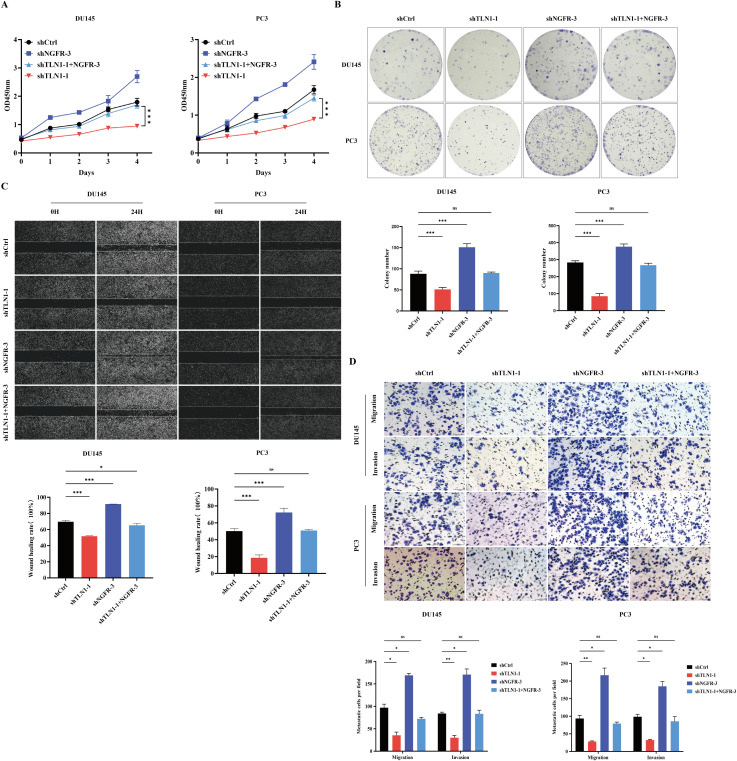
The existence of NGFR is essential for the TLN1-induced regulation of CRPC. **(A)** The cell proliferation ability of CRPC cell lines DU145 and PC3 with mere NGFR knockdown, mere TLN1 knockdown, or both, was evaluated by CCK-8 assay. **(B)** The colony formation ability of DU145 and PC3 cells with mere NGFR knockdown, mere TLN1 knockdown, or both, was assessed by colony formation assay. **(C)** The migration and wound closure capacity of DU145 and PC3 cells with mere NGFR knockdown, mere TLN1 knockdown, or both, was assessed by Wound healing assay. **(D)** The migration and invasion capacity of DU145 and PC3 cells with mere NGFR knockdown, mere TLN1 knockdown, or both, was assessed by Transwell assay. Data were presented as mean with standard deviation. "ns" indicates a P-value > 0.05. *P < 0.05, **P < 0.01 and ***P < 0.001.

To investigate whether NGFR is involved in TLN1-mediated regulation of CRPC, we introduced shNGFR-3 into DU145 and PC3 cells with or without TLN1 knockdown, establishing four experimental groups. Evaluation through CCK-8 assay, colony formation assay, wound healing assay, and Transwell assay demonstrated that NGFR knockdown significantly reversed the suppressive effects on malignant phenotypes induced by TLN1 knockdown in CRPC cells (**Figures 6A–D**). These results suggest that TLN1 likely promotes the progression of CRPC by regulating NGFR.

## Discussion

PCa is one of the most common malignant tumors in men worldwide, and CRPC, as an advanced stage of the disease, presents limited clinical treatment options with a high tendency for therapeutic resistance. Therefore, identifying new therapeutic targets and elucidating underlying mechanisms hold significant clinical importance. Initially, we employed MS to compare the peptide expression profiles in serum samples from patients with HSPC and those with CRPC. The analysis revealed that TLN1 was significantly upregulated in the serum of CRPC patients. Further validation using immunohistochemistry, qPCR, and Western blot on tissue samples consistently confirmed elevated TLN1 expression in PCa tissues. This finding aligns with previous reports by Xu et al., which indicated a positive correlation between TLN1 expression and both malignancy and lymph node metastasis in PCa (29), suggesting that TLN1 may play an important role in PCa progression.

TLN1 is a crucial cytoskeletal protein involved in integrin activation, cell adhesion, and migration (14, 31–37). Integrins are key mediators of cell-extracellular matrix interactions, regulating cell survival, proliferation, migration, and signal transduction (38, 39). In this study, we demonstrated that knockdown of TLN1 significantly inhibited the proliferation, colony formation, migration, and invasion, and promoted apoptosis in CRPC cell lines DU145 and PC3. Moreover, it markedly suppressed tumor growth in a nude mouse xenograft model. The *in vivo* experiments in this study primarily employed a subcutaneous xenograft model. While this model offers advantages in operability and reproducibility, it lacks an organ-specific microenvironment and cannot fully recapitulate the tumor-stromal interactions or spontaneous metastatic process of CRPC, thereby limiting the interpretability of the results regarding the simulation of the natural disease progression. Due to the high technical complexity, extended modeling time, and lower success rates associated with orthotopic and metastatic models, they were not established in the present study. Future research will utilize more clinically relevant models to further validate the role of TLN1 in tumor invasion and metastasis.

These results collectively clarify the promoting effect of TLN1 on the malignant phenotype of CRPC both *in vitro* and *in vivo*. Notably, TLN1 knockdown also led to upregulation of the epithelial marker E-cadherin and downregulation of the mesenchymal marker N-cadherin, suggesting that TLN1 may regulate tumor invasiveness by inhibiting the EMT process.

Although this study did not investigate the role of TLN1 in PCa bone metastasis, previous research by Jin et al. demonstrated that cdk5-mediated phosphorylation of TLN1 promotes bone metastasis in PCa through activation of β1 integrin (19). Considering the central role of TLN1 in cell adhesion and migration, along with reported associations between TLN1 and metastasis in various cancers (18, 20, 23, 27, 28, 40–45), we speculate that TLN1 may play a significant regulatory role in the metastatic process of PCa, particularly in more aggressive subtypes such as CRPC.

To further elucidate the molecular mechanisms by which TLN1 regulates CRPC, we performed transcriptome sequencing analysis on DU145 cells with TLN1 knockdown. The results showed that TLN1 knockdown led to differential expression of 147 genes, among which the NGFR was significantly upregulated. This finding was subsequently validated at both mRNA and protein levels. Furthermore, molecular docking and Co-IP experiments confirmed an interaction between TLN1 and NGFR. NGFR is a member of the neurotrophin receptor family. In PCa, multiple studies support a tumor-suppressive role for NGFR. NGFR expression shows a negative correlation with the malignant progression of prostate epithelial cells (46–48), and increased NGFR expression and activity enhance azacytidine-induced cell death in PCa cells, which aligns with our findings regarding NGFR’s role in CRPC. Our analysis of the public GEPIA2 database also revealed that NGFR expression is significantly lower in PCa tissues compared to adjacent normal tissues, further supporting its potential role as a tumor suppressor. The tumor-suppressive effect of TLN1 knockdown may be mediated through the regulation of NGFR. Functional rescue experiments demonstrated that knocking down NGFR in the context of TLN1 knockdown partially reversed the inhibitory effects of TLN1 loss on cell proliferation, migration, and invasion. This suggests that TLN1 may promote the malignant progression of CRPC by suppressing NGFR expression or activity. We also investigated the effects of TLN1 knockdown on the classical PI3K-AKT, MAPK, and NF−κB signaling pathways. TLN1 knockdown specifically inhibited AKT phosphorylation without affecting PI3K phosphorylation, indicating that TLN1 regulates the PI3K-AKT pathway primarily at the level of AKT activation. Moreover, TLN1 knockdown reduced the phosphorylation of ERK and JNK. In contrast, no significant effect was observed on the NF−κB pathway. These results suggest that TLN1 can influence the malignant progression of CRPC through the PI3K−AKT and MAPK signaling pathways. Although the precise regulatory mechanism remains to be fully elucidated, we have discovered a direct interaction between TLN1 and NGFR and preliminarily revealed the functional importance of the TLN1/NGFR axis in CRPC.

This study provides new potential therapeutic targets for CRPC. Current treatments for CRPC primarily focus on the androgen receptor signaling axis, but drug resistance becomes increasingly prominent as the disease progresses. The discovery of the TLN1/NGFR axis, particularly the high expression of TLN1 in CRPC and its cancer-promoting function, suggests that inhibiting TLN1 could represent a novel therapeutic strategy. Small-molecule inhibitors or antibody drugs targeting TLN1 may inhibit tumor progression by relieving the suppression of NGFR and restoring its tumor-suppressive function. Additionally, TLN1 expression levels could serve as a biomarker for predicting CRPC progression or treatment response.

This study also has several limitations. First, due to increased awareness of early intervention and treatment, coupled with diagnostic challenges for CRPC patients, obtaining clinical samples from CRPC patients is difficult, and thus validation of TLN1 in CRPC clinical samples was not performed. Second, the specific molecular mechanisms by which TLN1 regulates NGFR have not been deeply investigated. Third, the role of the TLN1/NGFR axis in CRPC drug resistance has not been validated. Finally, although this study confirmed that TLN1 knockdown inhibits tumor growth *in vivo*, its effects have not been evaluated in spontaneous metastasis or bone metastasis models.

## Conclusion

In conclusion, our study identifies TLN1 as a promoter of CRPC progression. We demonstrate that TLN1 exerts its oncogenic effects, at least in part, by interacting with and upregulating the tumor suppressor NGFR. Silencing TLN1 inhibits malignant phenotypes by upregulating NGFR and regulates PI3K-AKT and MAPK signaling pathways in CRPC cell lines. These findings reveal the TLN1/NGFR interaction as a novel and promising therapeutic target for combating CRPC. Targeting this axis may provide a new strategy to overcome treatment resistance and improve outcomes for patients with advanced PCa.

## Data Availability

The datasets presented in this study can be found in online repositories. The names of the repository/repositories and accession number(s) can be found in the article/**Supplementary Material**.
