# Supplementary material for: TLN1 interacts with NGFR and suppresses the development of castration-resistant prostate cancer by upregulating NGFR
Source: Front Immunol. 2026 Apr 23;17:1802129. doi: 10.3389/fimmu.2026.1802129 (PMC13149361; doi:10.3389/fimmu.2026.1802129)

Supplementary figure1： WB original figure

Figure 2G

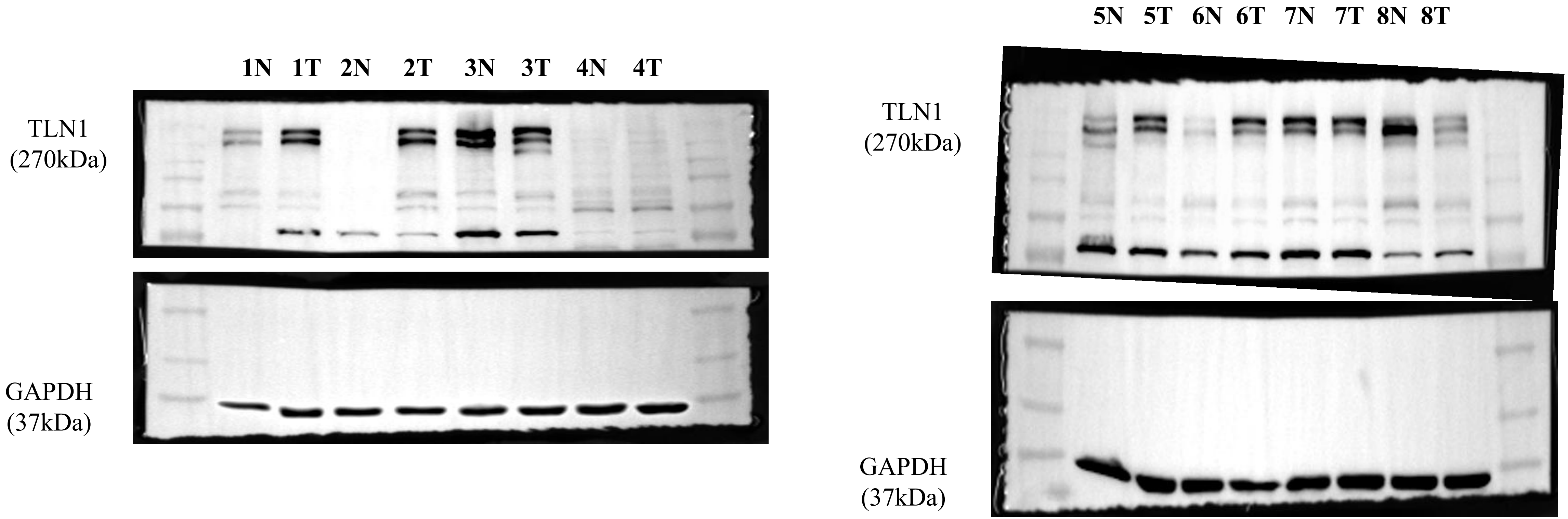

Figure 2G

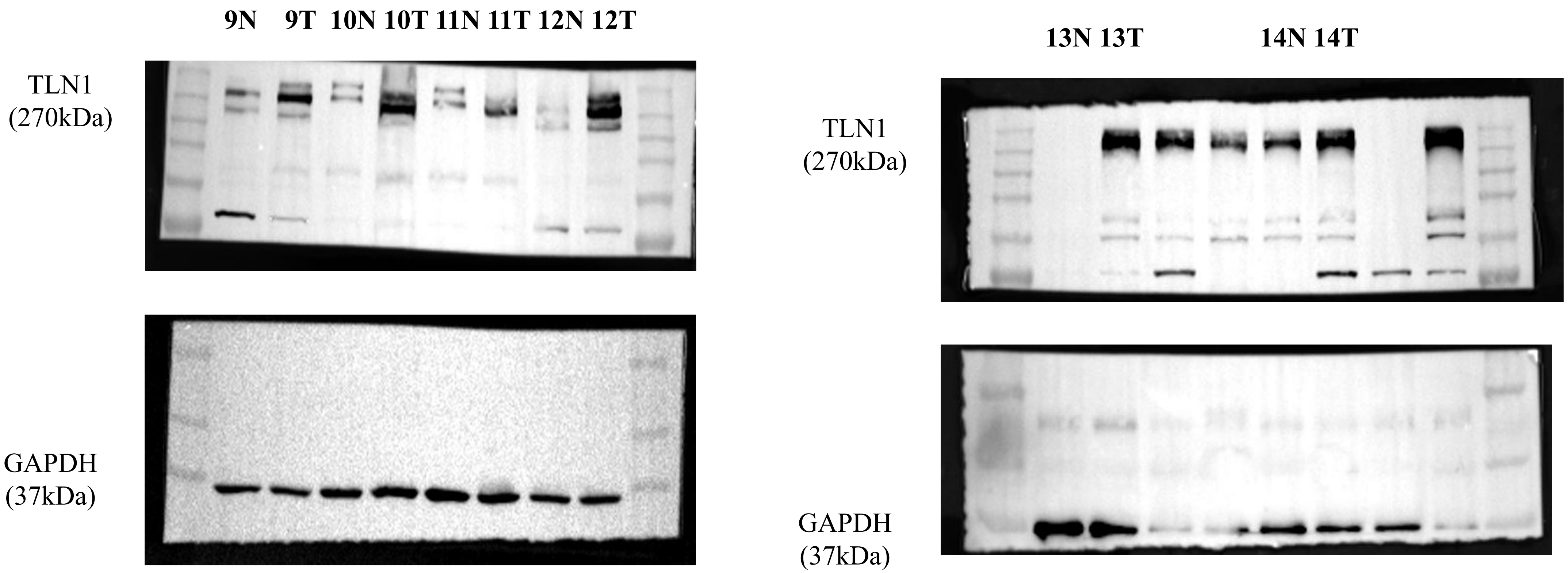
9N 9T 10N 10T 11N 11T 12N 12T
TLN1
(270kDa)
GAPDH
(37kDa)
13N 13T
14N 14T
TLN1
(270kDa)
GAPDH
(37kDa)

Figure 2G

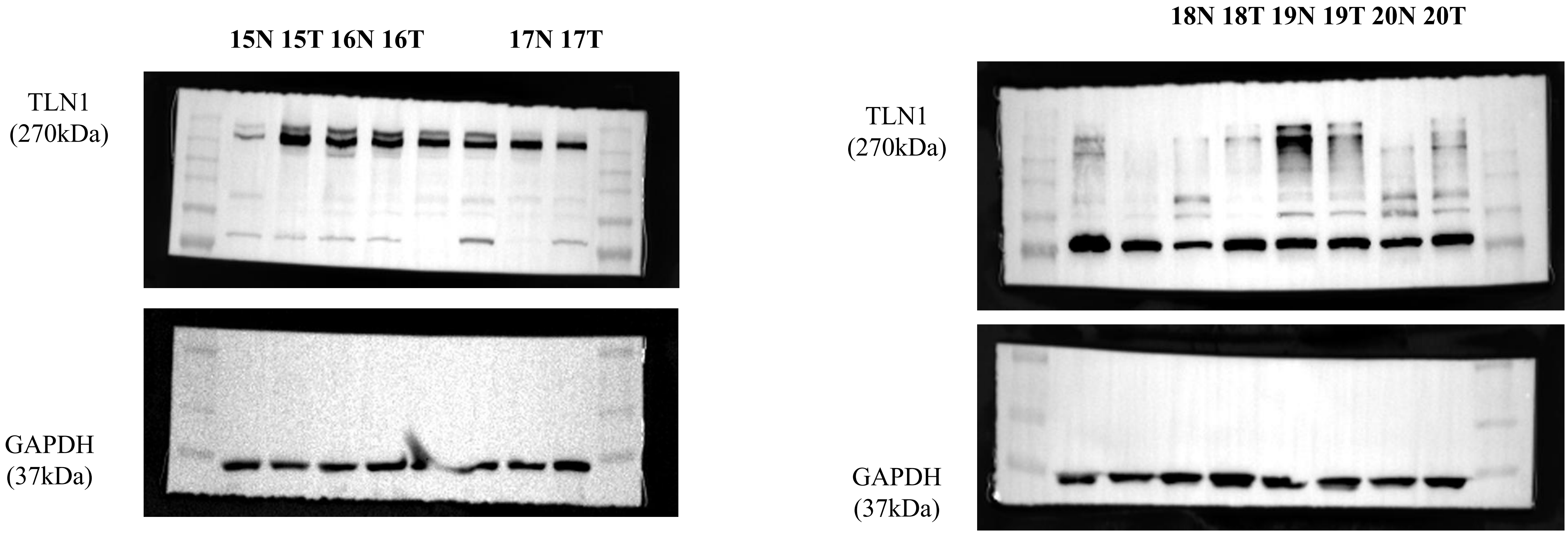
15N 15T 16N 16T 17N 17T
TLN1
(270kDa)
GAPDH
(37kDa)
18N 18T 19N 19T 20N 20T
TLN1
(270kDa)
GAPDH
(37kDa)

Figure 2J

TLN1
(270kDa)

GAPDH
(37kDa)

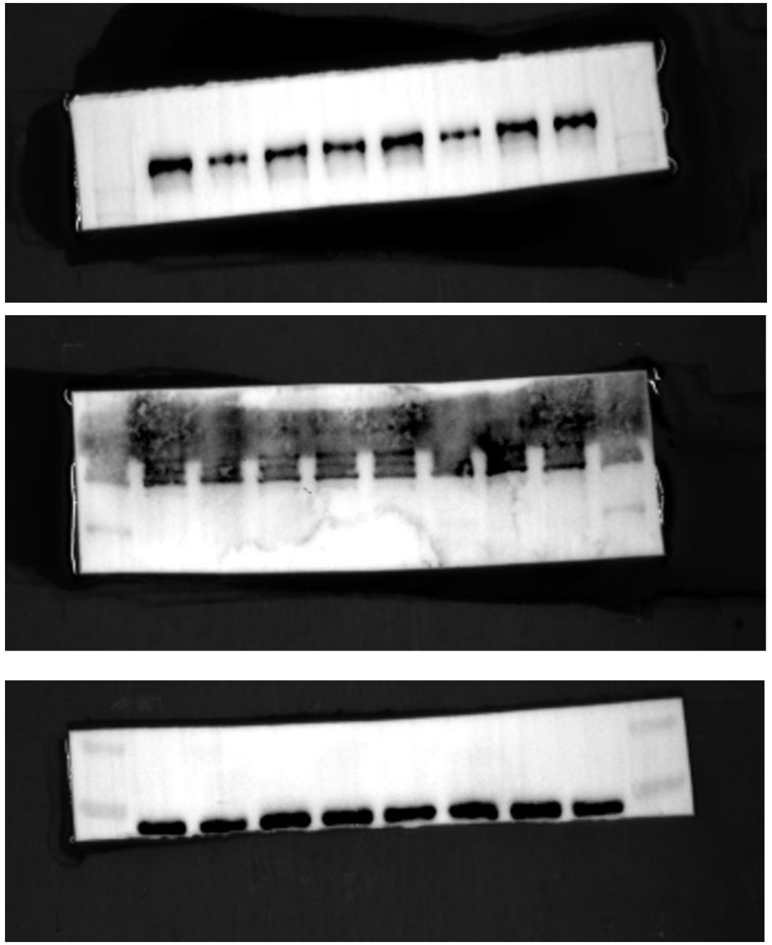

Figure 3E

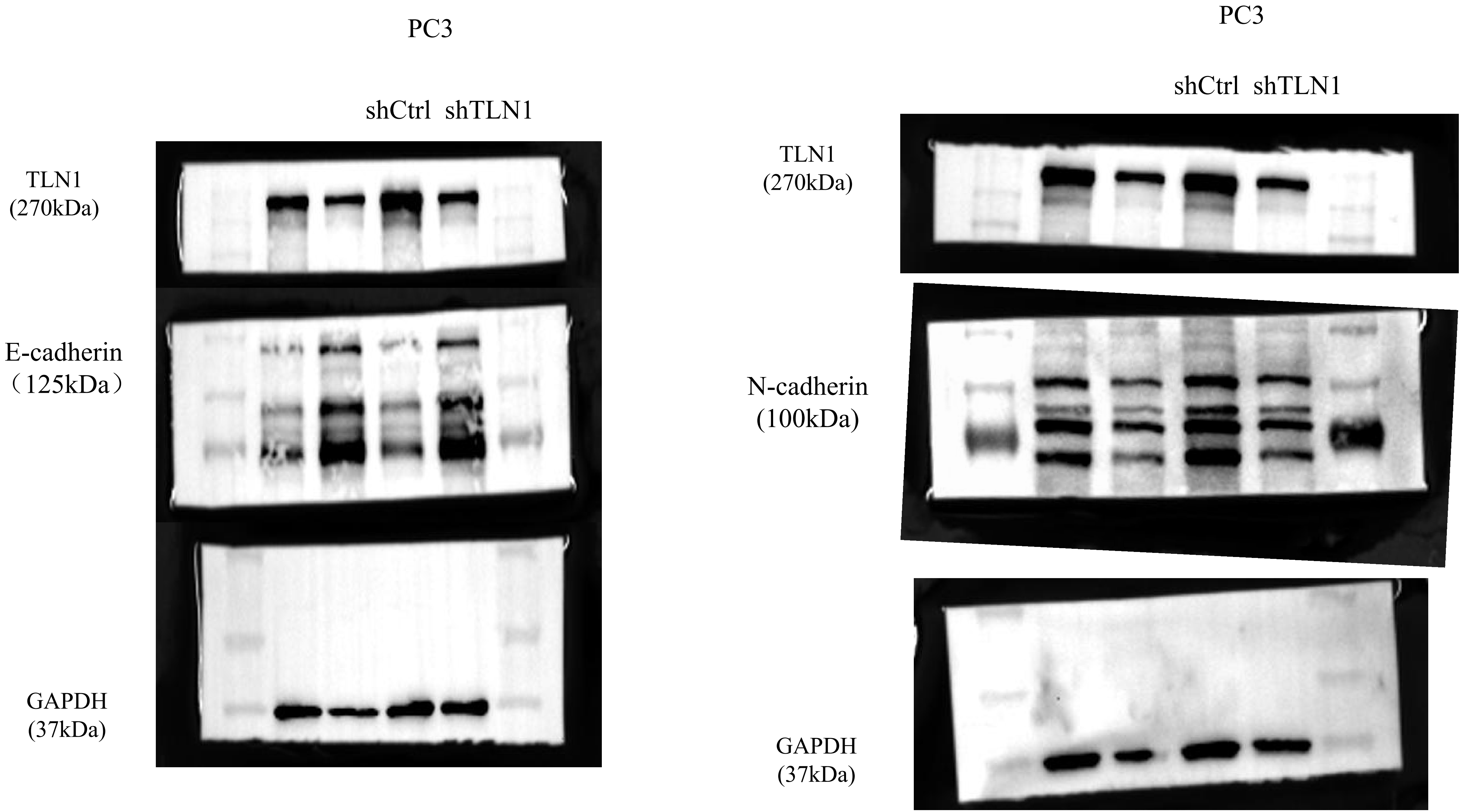
PC3
shCtrl shTLN1
TLN1
(270kDa)
E-cadherin
（125kDa）
GAPDH
(37kDa)
PC3
shCtrl shTLN1
TLN1
(270kDa)
N-cadherin
(100kDa)
GAPDH
(37kDa)

Figure 3E

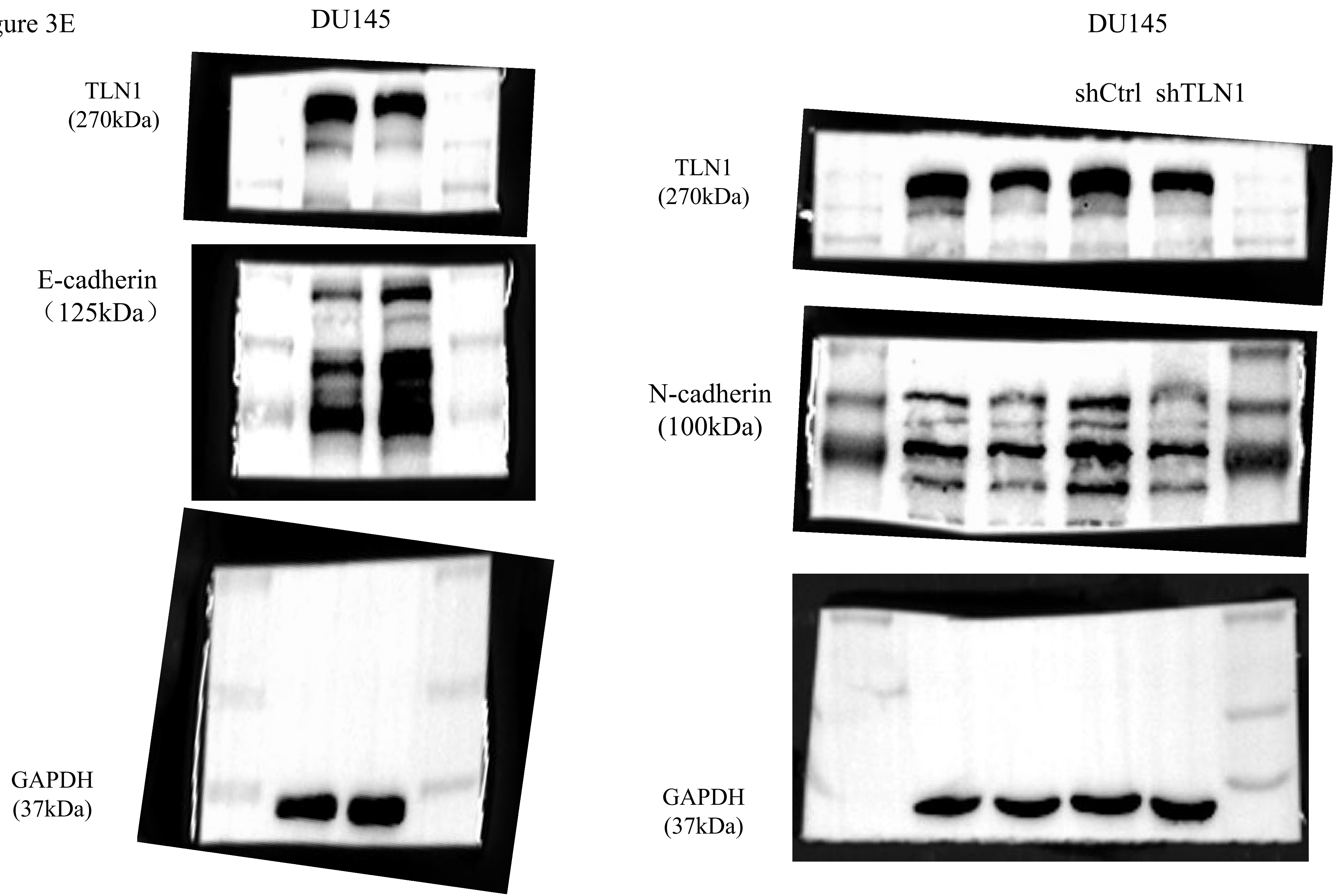
DU145
TLN1
(270kDa)
E-cadherin
（125kDa）
GAPDH
(37kDa)
DU145
shCtrl shTLN1
TLN1
(270kDa)
N-cadherin
(100kDa)
GAPDH
(37kDa)

Figure 4F

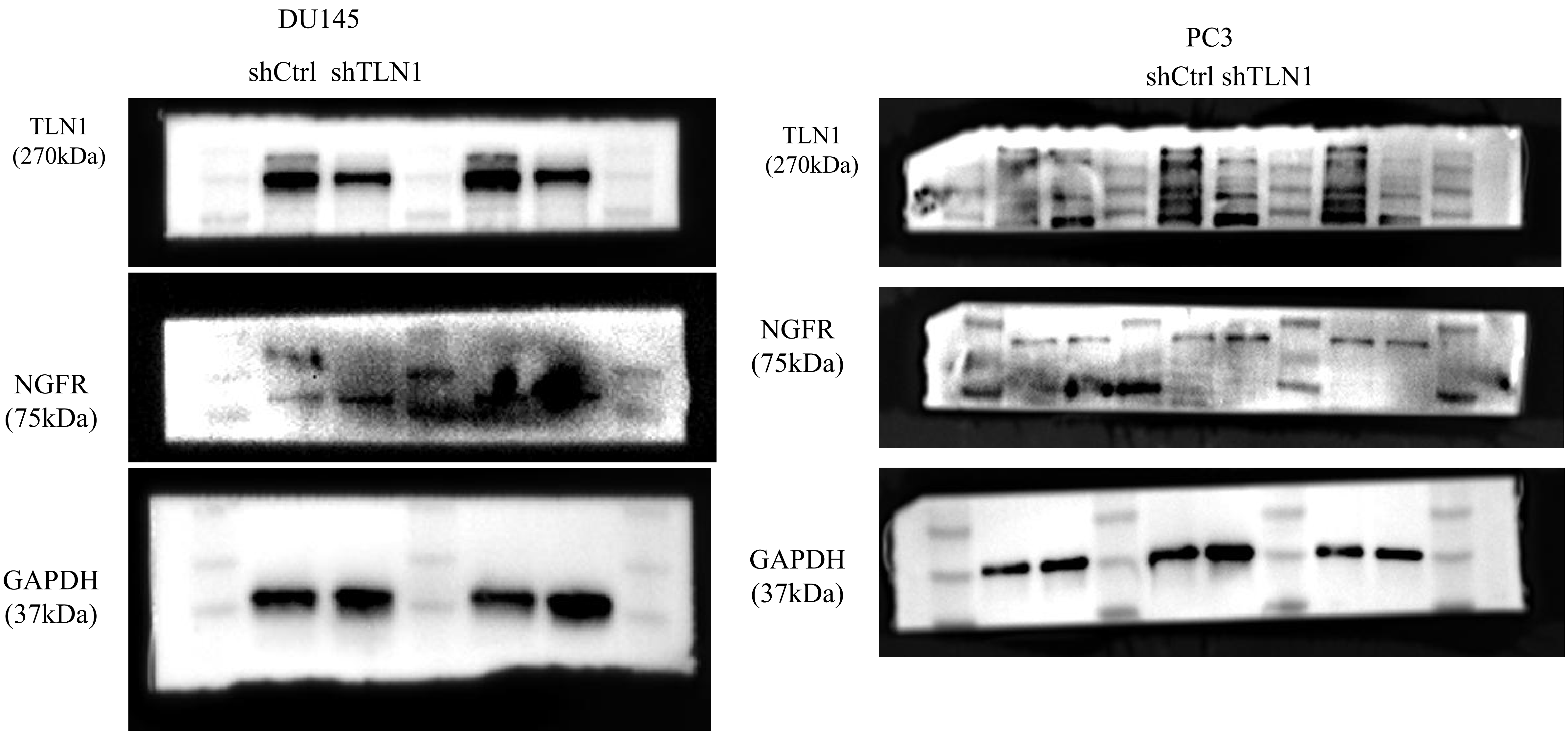

DU145
shCtrl shTLN1
TLN1
(270kDa)
NGFR
(75kDa)
GAPDH
(37kDa)
PC3
shCtrl shTLN1
TLN1
(270kDa)
NGFR
(75kDa)
GAPDH
(37kDa)

Figure 4G

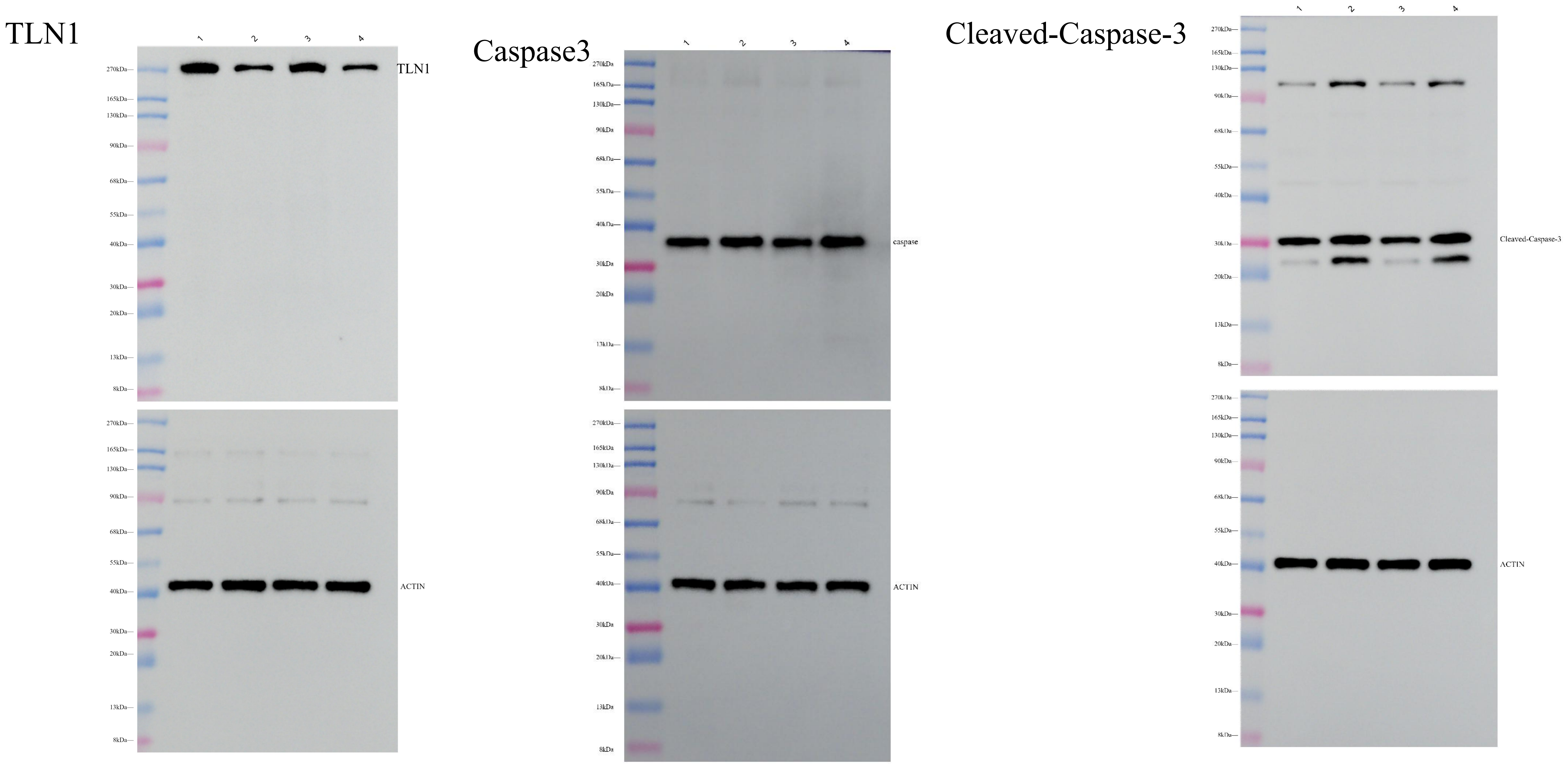
TLN1
1
2
3
4
TLN1
ACTIN
Caspase3
1
2
3
4
caspase
ACTIN
Cleaved-Caspase-3
1
2
3
4
Cleaved-Caspase-3
ACTIN

Figure 4G

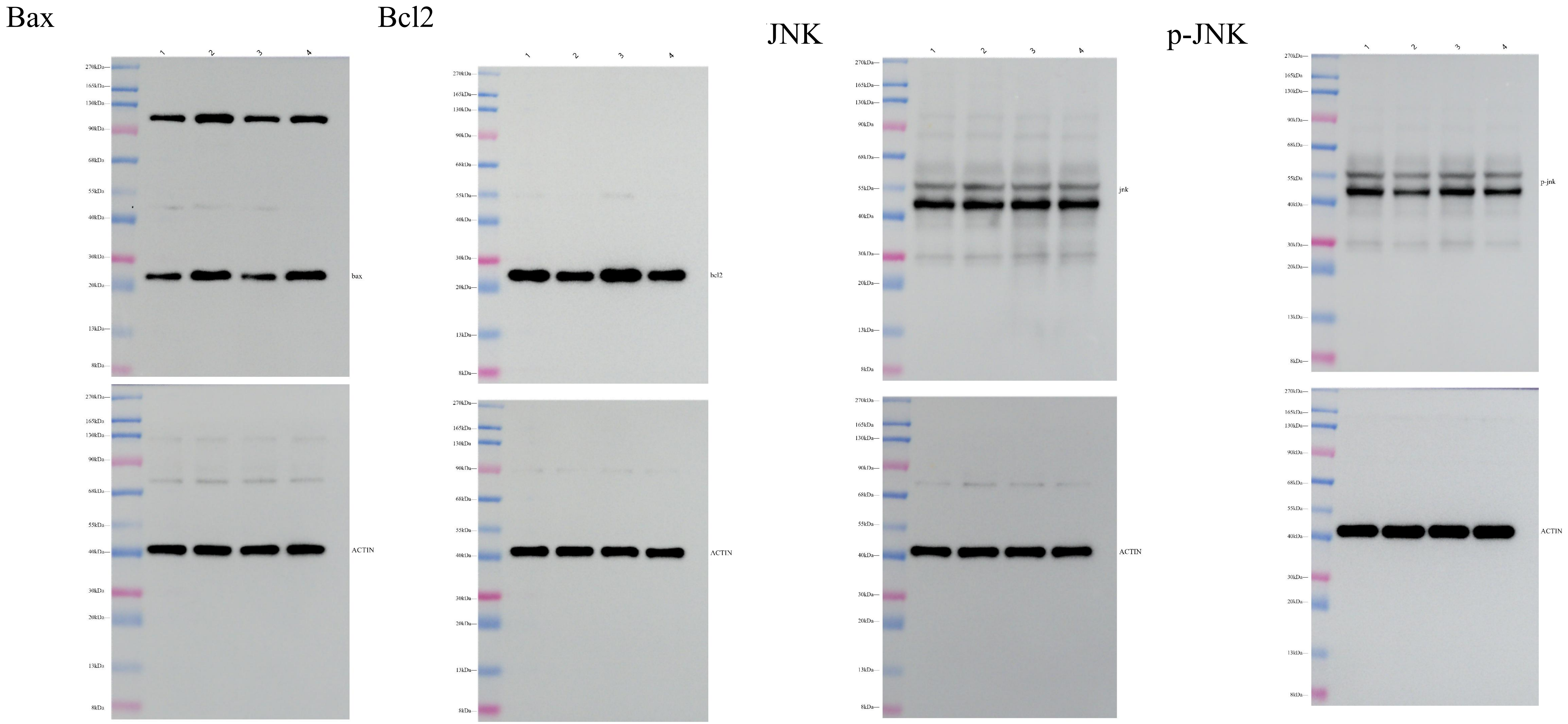

Bax
Bcl2
JNK
p-JNK
1
2
3
4
270kDa
165kDa
130kDa
90kDa
68kDa
55kDa
40kDa
30kDa
20kDa
13kDa
8kDa
bax
bcl2
jnk
p-jnk
ACTIN

Figure 4G

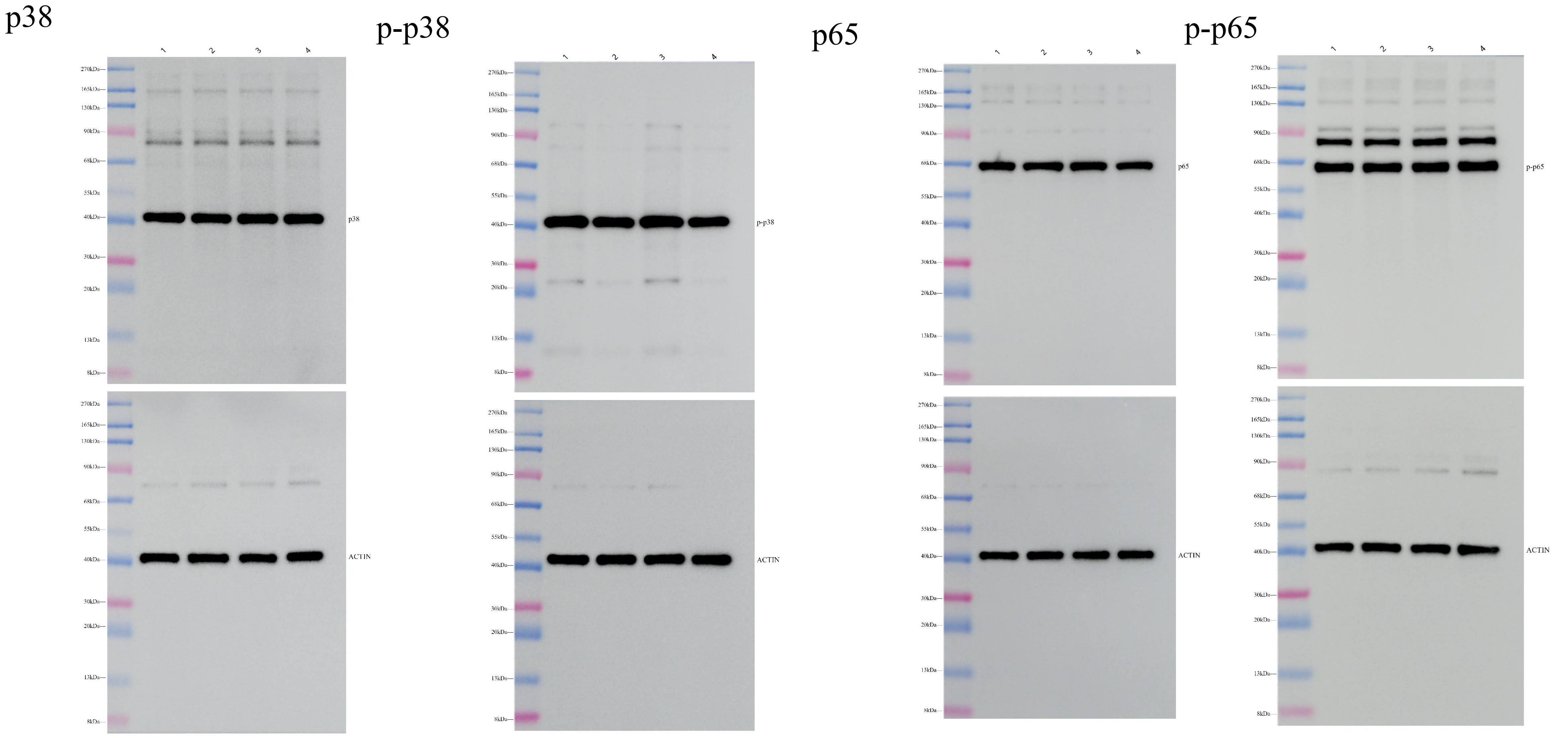
p38
p-p38
p65
p-p65
1
2
3
4
270kDa
165kDa
130kDa
90kDa
68kDa
55kDa
40kDa
30kDa
20kDa
13kDa
8kDa
p38
p-p38
p65
p-p65
ACTIN

Figure 4G

PI3K

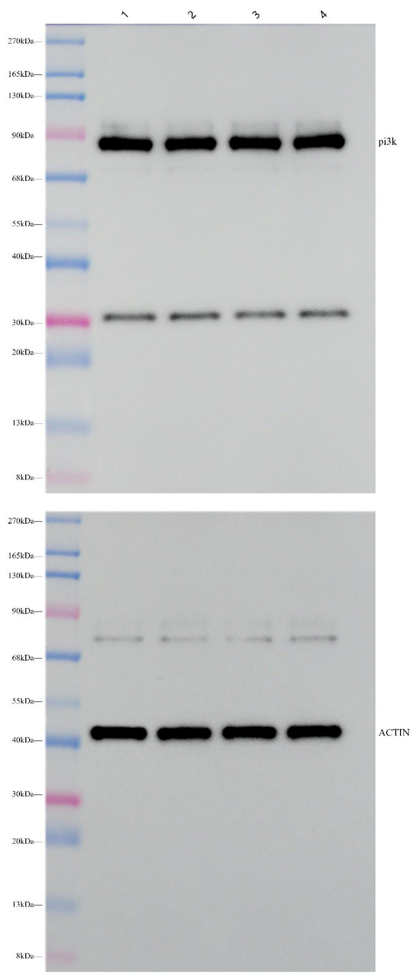

p-PI3K

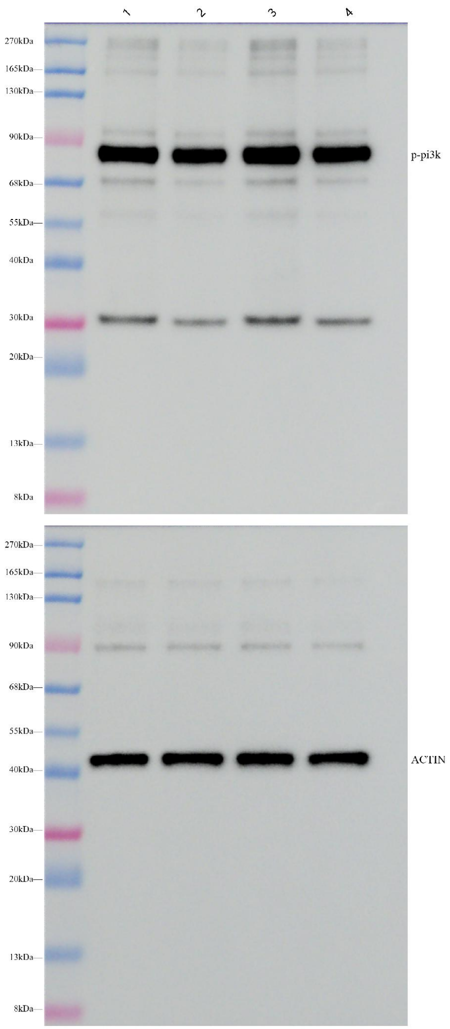

AKT

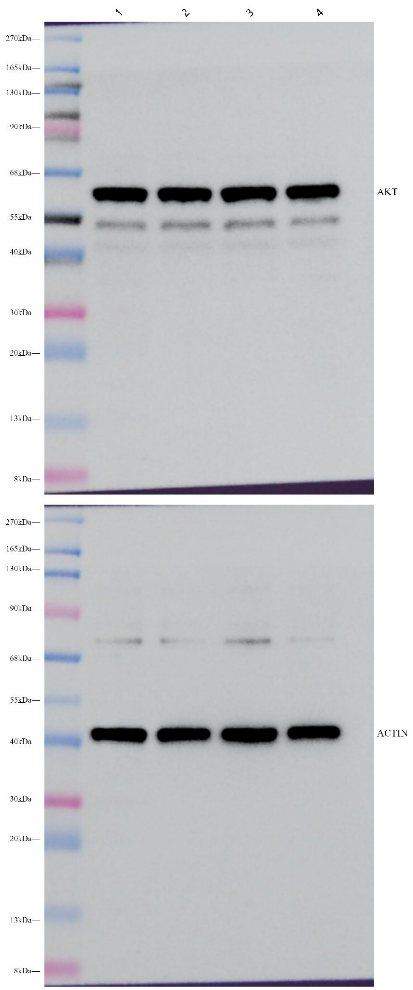

p-AKT

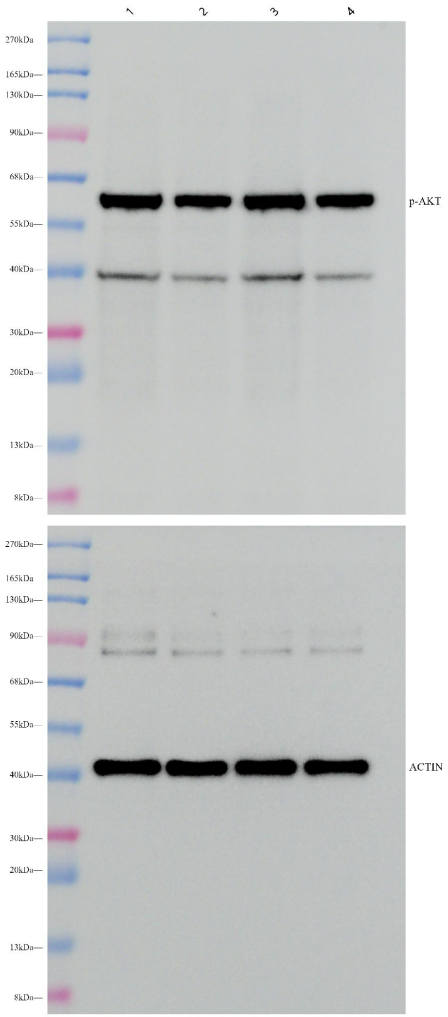

Figure 4G

ERK

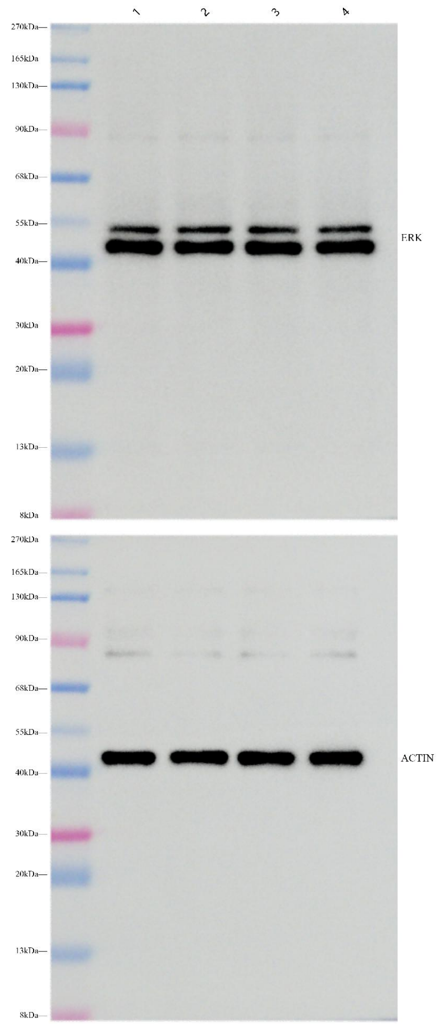

p-ERK

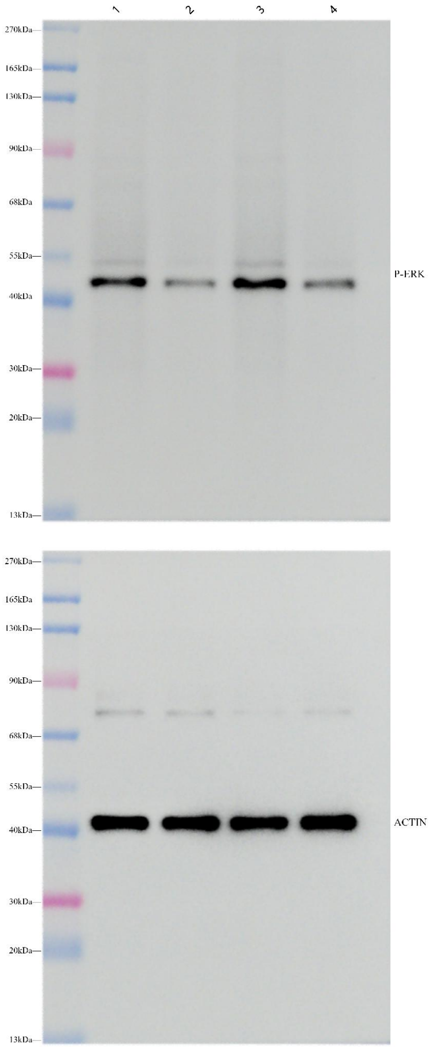

Figure 4I

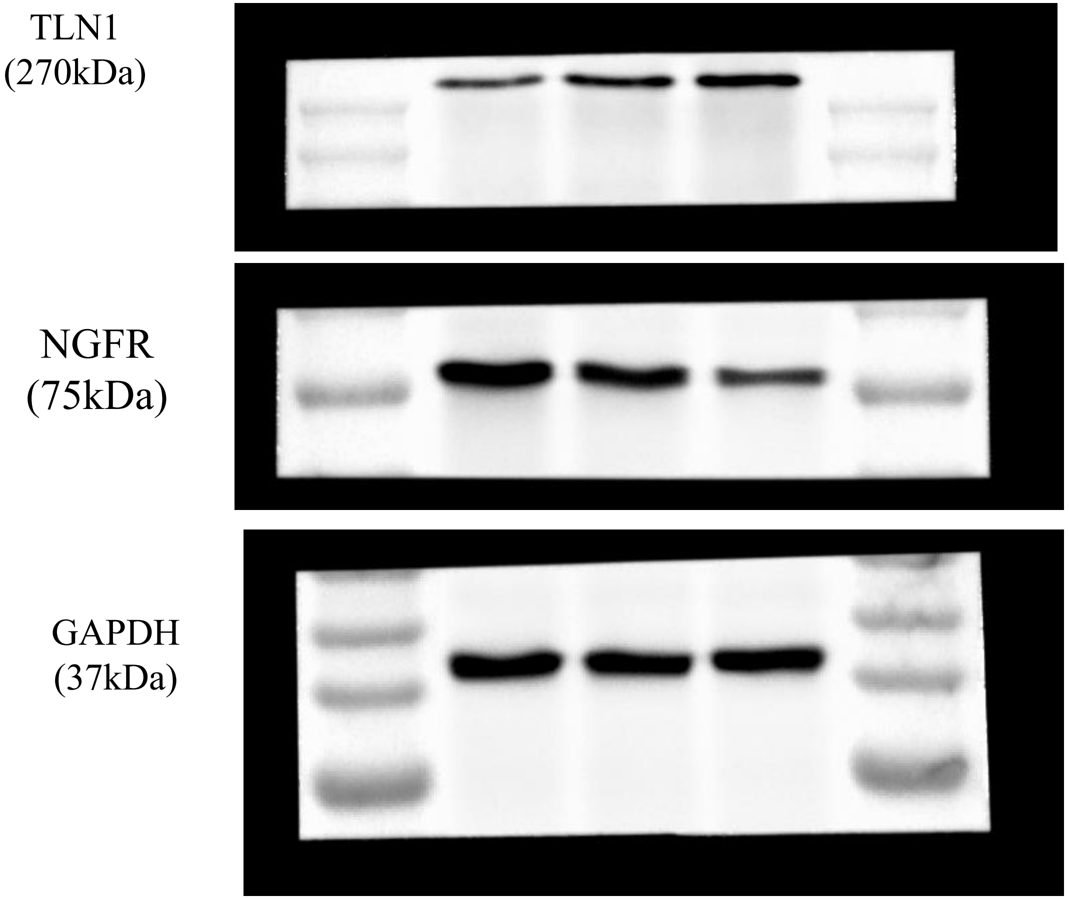

Figure 4L

TLN1
(270kDa)

NGFR
(75kDa)

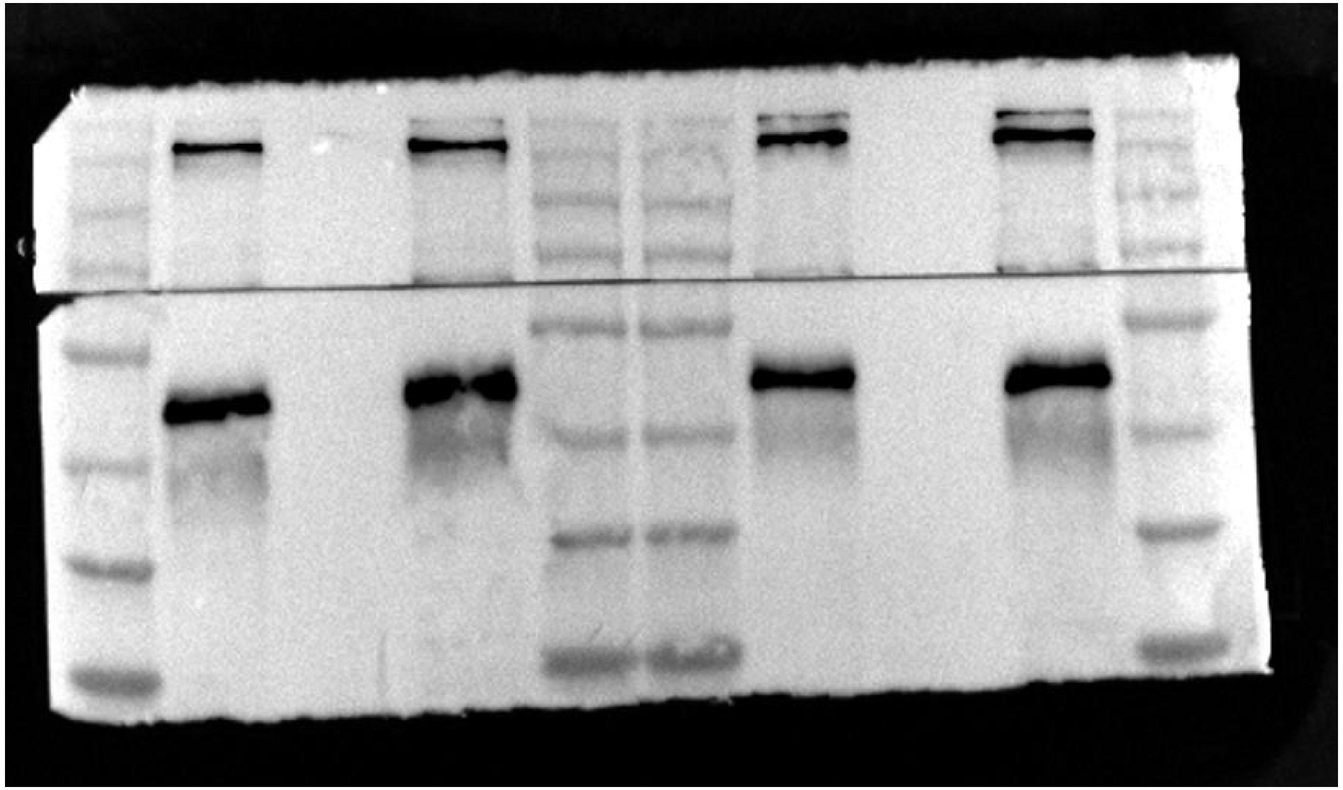

Figure 4N

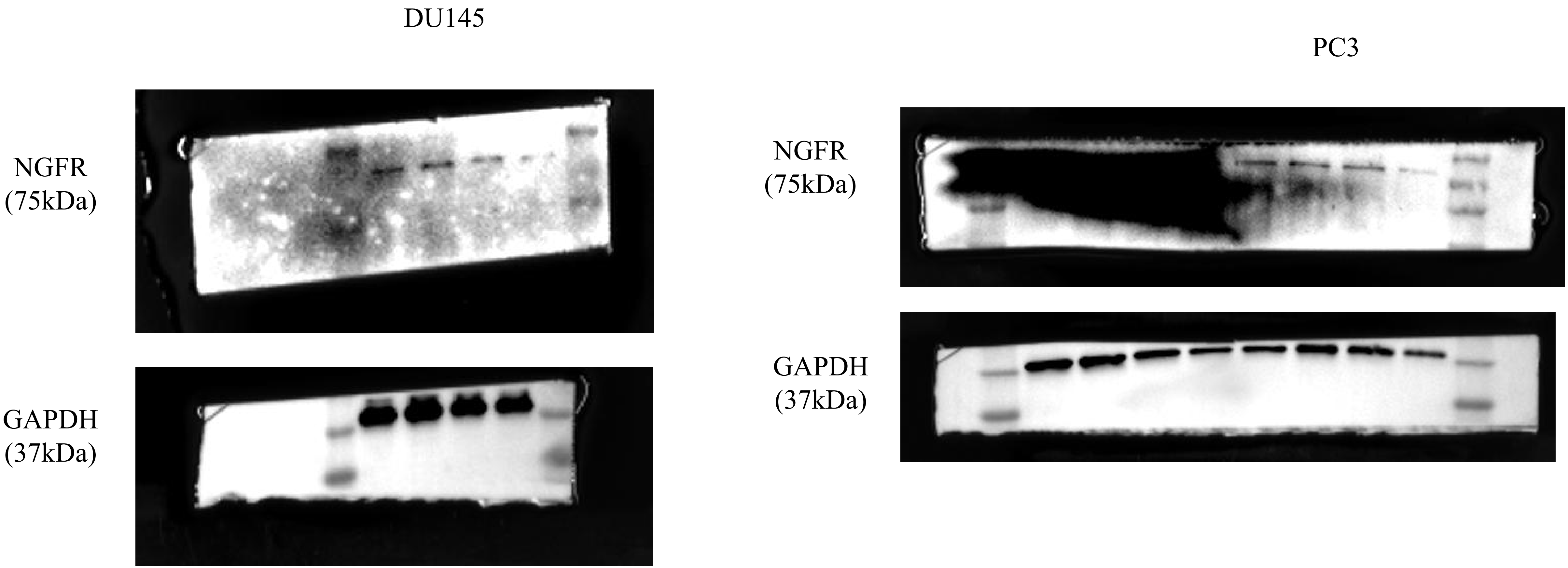

Supplement: Supplementary file 1 [file DataSheet1.pdf]
