# Supplementary material for: TLN1 interacts with NGFR and suppresses the development of castration-resistant prostate cancer by upregulating NGFR
Source: Front Immunol. 2026 Apr 23;17:1802129. doi: 10.3389/fimmu.2026.1802129 (PMC13149361; doi:10.3389/fimmu.2026.1802129)
Supplement: Supplementary file 2 [file Table1.docx]

**Supplementary table 1** **Sequences of primers used for qPCR.**

| **mRNA** | **Forward Primer** | **Reverse Primer** |
| --- | --- | --- |
| TLN1 | ACT TAC GGT GTC TCC TTC TTC CTG | GTC TTC TCA TCC ACT CGC ATC AC |
| NGFR | CTA CCA GCC CGA GCA CAT AG | CTG CAC AGA CTC TCC ACG AG |
| GAPDH | TGACTTCAACAGCGACACCCACACCCTGTTGCTGTAGCCAAA | TGACTTCAACAGCGACACCCACACCCTGTTGCTGTAGCCAAA |

**Supplementary table 2 Plasmid Sequences**

| **Plasmid name** | **Sequences** |  |
| --- | --- | --- |
| shCtrl | TTCTCCGAACGTGTCACGT |  |
| shTLN1-1 | CCTTCGTGGATTACCAAACAA |  |
| shTLN1-2  shTLN1-3  shNGFR-1  shNGFR-2  shNGFR-3 | CGCATTGGCATCACCAATCAT  CCCAGAGTATTAACGCTCCAA  GGAGGTGCCAAGGAGGCATGC  GGACAGCCAGAGCCTGCATGA  GCACCACCGACAACCTCATCC |  |
